# Activation of Piezo1 by intracranial hypertension induced neuronal apoptosis via activating hippo pathway

**DOI:** 10.1111/cns.14872

**Published:** 2024-09-27

**Authors:** Jia Zeng, Zhen Fang, Jiajia Duan, Zichen Zhang, Yunzhi Wang, Yiping Wang, Lei Chen, Jikai Wang, Fei Liu

**Affiliations:** ^1^ Department of Neurosurgery, Guangdong Provincial Key Laboratory of Biomedical Imaging The Fifth Affiliated Hospital of Sun Yat‐sen University Zhuhai China

**Keywords:** apoptosis, hippo pathway, intracranial hypertension, neuron, Piezo1, subarachnoid hemorrhage

## Abstract

**Aim:**

Most of the subarachnoid hemorrhage (SAH) patients experienced the symptom of severe headache caused by intracranial hypertension. Piezo1 is a mechanosensitive ion channel protein. This study aimed to investigate the effect of Piezo1 on neurons in response to intracranial hypertension.

**Methods:**

The SAH rat model was performed by the modified endovascular perforation method. Piezo1 inhibitor GsMTx4 was administered intraperitoneally after SAH induction. To investigate the underlying mechanism, the selective Piezo1 agonist Yoda1, Piezo1 shRNA, and MY‐875 were administered via intracerebroventricular injection before SAH induction. In vitro, we designed a pressurizing device to exclusively explore the effect of Piezo1 activation on primary neurons. Neurons were pretreated with Piezo1 inhibition followed by intracranial hypertension treatment, and then apoptosis‐related proteins were detected.

**Results:**

Piezo1 inhibition significantly attenuated neuronal apoptosis and improved the outcome of neurological deficits in rats after SAH. The Hippo pathway agonist MY‐875 reversed the anti‐apoptotic effects of Piezo1 knockdown. In vitro, intracranial hypertension mimicked by the pressurizing device induced Piezo1 expression, resulting in Hippo pathway activation and neuronal apoptosis. The Hippo pathway inhibitor Xmu‐mp‐1 attenuated Yoda1‐induced neuronal apoptosis. In addition, the combination of hypertension and oxyhemoglobin treatment exacerbated neuronal apoptosis.

**Conclusions:**

Intracranial hypertension induced Piezo1 expression, neuronal apoptosis, and the Hippo pathway activation; the Hippo signaling pathway is involved in Piezo1 activation‐induced neuronal apoptosis in respond to intracranial hypertension. Primary neurons treated with intracranial hypertension and oxyhemoglobin together can better characterize the circumstance of SAH in vivo, which is contributed to construct an ideal in vitro SAH model.

## Graphical abstract

Here, we found that Piezo1 inhibition attenuated neuronal apoptosis and improved the outcome of neurological deficits in rats after subarachnoid hemorrhage. Activation of Piezo1 by intracranial hypertension induced neuronal apoptosis via activating the Hippo pathway in primary neurons. Furthermore, this study incorporated the variables of oxyhemoglobin and intracranial hypertension into the cultivation of neurons, resulting in a compounded impact on neuronal apoptosis.

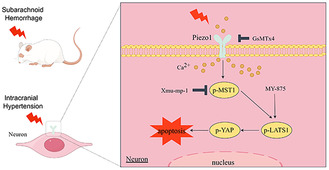

## 1 INTRODUCTION

Subarachnoid hemorrhage (SAH) is a life‐threatening cerebrovascular disease, which is a subtype of hemorrhagic stroke with high morbidity and mortality.^1^, ^2^, ^3^ SAH refers to a result of bleeding into the subarachnoid space, so it is always associated with sudden‐onset headache.^4^, ^5^ As the disease progresses, intracranial hypertension is usually markedly elevated and even causing cerebral herniation in SAH patients.^6^ Previous studies mainly focus on secondary brain injuries caused by hemoglobin toxicity, oxidative stress, and inflammation.^7^, ^8^, ^9^, ^10^ A few studies reported the effects of primary brain injury, intracranial hypertension, on the outcome of neurological function. The occurrence of intracranial hypertension is prevalent and has significant implications for adverse outcomes in individuals diagnosed with SAH. Therefore, the management of intracranial hypertension holds paramount importance in clinical practice.^11^ In this study, our research efforts were directed towards examining the mechanisms implicated in intracranial hypertension.

Neuronal apoptosis is a key pathophysiologic mechanism in the process of SAH. Numerous studies elucidated the pivotal role of neuronal apoptosis in SAH.^5^, ^12^, ^13^ Friedrich et al. documented neuronal apoptosis occurring 10 min after SAH in rats, representing the earliest manifestation of cell death in early brain injury.^13^ It is worth stressing that neuronal apoptosis can be found in nearly all kinds of central nervous system diseases including Alzheimer's disease and Parkinson's disease.^14^, ^15^ Neurological function deficits in motion and memory can be observed when numerous neurons are lost.^16^ Our team proved that the neurological function was improved by attenuating neuronal apoptosis and enhancing neural stem cells differentiation towards neurons in mice after SAH.^7^, ^8^ The mitigation of intracranial hypertension is closely related to the improvement of neurological deficits in SAH patients, but the association between intracranial hypertension and neuronal apoptosis remains uncertain.^17^, ^18^

Piezo1, a kind of mechanosensitive ion channel protein, can convert extracellular mechanical stimulus into intracellular electrical signals.^19^ Studies shown that Piezo1 inhibition exerts neuroprotective effects by attenuating demyelination, modulating microglial immune responses. A study reported that Piezo1 inhibition can attenuate intracerebral hemorrhage‐induced demyelination by alleviating oligodendrocyte apoptosis.^20^ Furthermore, migration toward amyloid β‐protein was enhanced in microglia with Piezo1 deficiency, and levels of pro‐inflammatory cytokines produced upon stimulation by lipopolysaccharide were decreased.^21^ In addition, the factor of mechanical forces is closely related to the developing brain; the involvement of mechanosensitive ion channels in detecting local tissue stiffness is crucial for guiding neuronal growth.^22^ Taken together, the mechanical characteristic of Piezo1 is strongly associated with central nervous system diseases. However, the relationship between Piezo1 and mechanical stimulus has not been explored in SAH.

The Hippo pathway, an evolutionarily conserved signaling pathway in mammals, plays a crucial role in organ development and oncogenesis. Activation of the Hippo pathway induces cell apoptosis, whereas inhibition of this pathway promotes cell proliferation.^23^ A previous study reported that inhibition of MST1 phosphorylation, a core element and protein kinase of the Hippo pathway, attenuated neuronal apoptosis, and improved neurological deficits in rats following intracerebral hemorrhage.^24^ The Hippo pathway was discussed widely in all kinds of diseases, especially in carcinogenesis; Piezo1 has been shown to modulate the nuclear translocation of YAP in tumors, thereby facilitating tumor metastasis. The mechanosensitive protein Piezo1 is closely related to the Hippo‐YAP signaling pathway.

In this study, we investigated the role of Piezo1 in SAH in vivo. A pressurizing device was developed to exclusively explore the effect of intracranial hypertension on primary neurons. Our results demonstrated that Piezo1 inhibition caused anti‐apoptotic effect in rats after SAH. In addition, Piezo1 inhibition attenuated neuronal apoptosis caused by hypertension via inhibiting the Hippo signaling pathway in primary neurons.

## 2 MATERIALS AND METHODS

### 2.1 Animals and surgical induction of SAH

Male Sprague–Dawley rats of adult age, weighing between 250 and 280 g, were accommodated in a controlled environment with a consistent temperature of 25°C, regulated humidity, and subjected to a 12 h light and dark cycle. Additionally, rats were provided unrestricted availability of standard food and water. All the procedures in this experiment were approved by the Institutional Animal Care and Use Committee of the Fifth Affiliated Hospital of Sun Yat‐sen University and in accordance with the National Institutes of Health Guide for the Care and Use of Laboratory Animals (2021122102). All experiments were reported according to the Animal Research: Reporting In Vivo Experiments (ARRIVE) guidelines.

The subarachnoid hemorrhage rat model was executed using a modified endovascular perforation technique, as previously documented.^12^ Briefly, rats were anesthetized with 5% isoflurane and anesthesia was maintained with 3% isoflurane on a ventilator. The midline incision was made in the neck of rats. Subsequently, a meticulous dissection of the left common carotid artery, external carotid artery, and internal carotid artery was performed (Figures S1C). A 4–0 monofilament nylon suture was then carefully inserted into the left internal carotid artery, originating from the stump of the external carotid artery, until reaching the point of bifurcation between the anterior and middle cerebral arteries, where a noticeable resistance was encountered. Finally, experimenters inserted the nylon suture 3 mm advanced to perforate the bifurcation and withdrawn the suture immediately. Rats of the Sham group underwent identical procedures except the perforation. Finally, the skin incision in the neck was sutured. After waking up from anesthesia, rats were housed in heated cages.

### 2.2 Experimental design

**FIGURE 1:**
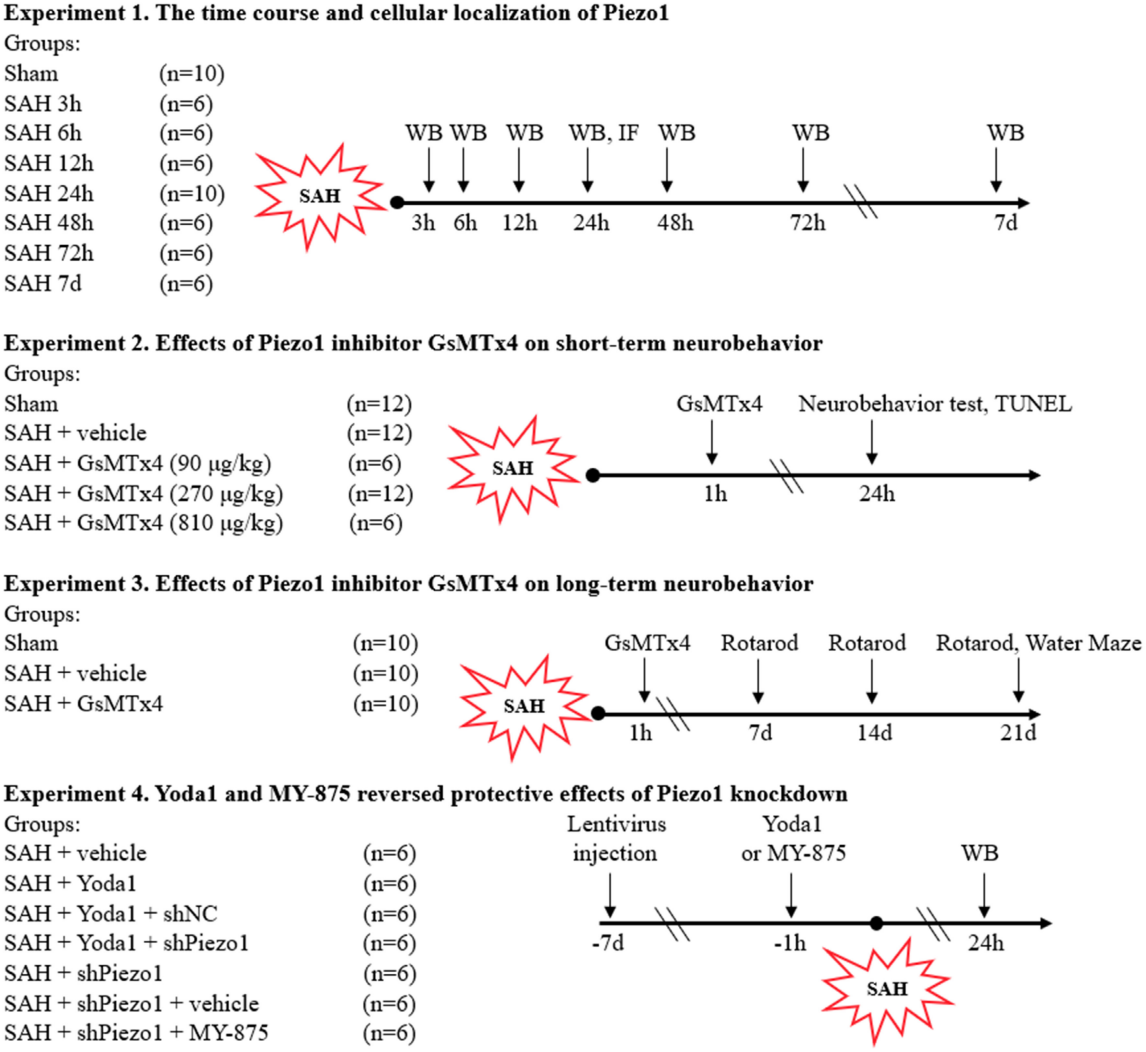
Experimental design and animal groups. IF, Immunofluorescence; SAH, subarachnoid hemorrhage; WB, Western blot.

The experimental design for the animal part has been shown in Figure 1.

#### 2.2.1 Experiment 1

To determine the time course and cellular localization of Piezo1 after SAH, a total of 48 rats were divided randomly into eight groups (*n* = 6): Sham and SAH 3 h, 6 h, 12 h, 24 h, 48 h, 72 h and 7 days. According to the different time points, rats were euthanized after SAH. Western blot was performed to detect the expression changes of Piezo1. Another eight rats in the Sham and SAH 24 h groups (*n* = 4) were used for double‐labeling immunofluorescence.

#### 2.2.2 Experiment 2

To evaluate the effects of Piezo1 inhibitor GsMTx4 on short‐term neurobehavior after SAH, a total of 30 rats were divided into five groups (*n* = 6): Sham, SAH + vehicle, SAH + GsMTx4 (90 μg/kg), SAH + GsMTx4 (270 μg/kg), SAH + GsMTx4 (810 μg/kg). The modified Garcia score and beam balance test were performed at 24 h after SAH. According to results of modified Garcia score and balance beam test, GsMTx4 (270 μg/kg) as the optimal drug concentration was used for TUNEL staining and subsequent long‐term neurological assessments. In addition to assess the effects of GsMTx4 on neuronal apoptosis, TUNEL staining was carried out at 24 h after SAH with another set of 18 rats divided into three groups (*n* = 6): Sham, SAH + vehicle, SAH + GsMTx4 (270 μg/kg).

#### 2.2.3 Experiment 3

To evaluate the neuroprotective effects of Piezo1 inhibitor GsMTx4 on long‐term neurobehavior after SAH, another 30 rats were randomly assigned into three groups (*n* = 10): Sham, SAH + vehicle, SAH + GsMTx4 (270 μg/kg). On days 7, 14, 21 and days 22–27 after SAH, we performed the rotarod beam test and Morris water maze test respectively.

#### 2.2.4 Experiment 4

To investigate the potential mechanism of Piezo1 inhibition mediated anti‐apoptotic effects after SAH, a total of 24 rats were randomly divided into four groups (*n* = 6): SAH + vehicle, SAH + Yoda1, SAH + Yoda1 + shNC, SAH + Yoda1 + shPiezo1. To further prove the pathway, another 18 rats were randomly divided into three groups (*n* = 6): SAH + shPiezo1, SAH + shPiezo1 + vehicle, SAH + shPiezo1 + MY‐875. Western blot was performed to detect the protein levels of apoptosis‐associated proteins and Hippo pathway components.

### 2.3 Drug administration

GsMTx4 (MedChem Express), a non‐selective inhibitor of Piezo1, was diluted in 10% dimethyl sulfoxide (DMSO). There different dosages (90 μg/kg, 270 μg/kg, 810 μg/kg) of GsMTx4 were prepared and GsMTx4 was administered intraperitoneally at 1 h after SAH induction. Rats in the SAH + vehicle group received an equal volume of 10% DMSO.

### 2.4 Intracerebroventricular injection

We performed intracerebroventricular injection (i.c.v.) as described previously.^7^, ^8^ In short, rats were placed in a stereotaxic apparatus under 3% isoflurane anesthesia. Then, experimenters drilled a cranial hole 1.0 mm posterior and 1.5 mm lateral to Bregma. Injection was performed via a 10 μL Hamilton syringe (Microliter 701, Hamilton Company). The experimenters inserted the syringe into the right ventricle through the cranial hole at a depth of 3.3 mm. The i.c.v. injection rate was 1.0 μL/min by a pump and the syringe was left in place for 5 min at the end of injection. The Piezo1 agonist Yoda1 (Tocris Bioscience) was prepared in DMSO at a concentration of 25 μm. The Hippo pathway agonist MY‐875 (GLPBIO) was prepared in DMSO at a concentration of 10 μm. A volume of 5.0 μL of Yoda1 or MY‐875 was administered via infusion 1 h prior to SAH induction. Lentiviruses for Piezo1 knockdown were produced by TSINGKE (Beijing TSINGKE Biotech Co., Ltd.): shPiezo1 (sense: GCTTCTACCTGCTGCTCTTTG). Five microliters of lentivirus with 9 × 10^9^ genomic copies of shPiezo1 were injected into the right ventricle. Seven days later, the rats underwent SAH surgery. Following removal of the syringe from the cranial hole, the hole was filled with bone wax.

### 2.5 SAH grade measurement

According to previously published articles,^7^, ^8^ the evaluation system is based on the amount of subarachnoid blood clots distributed in the six segments of the basal cistern: 0, no subarachnoid blood; 1, minimal subarachnoid blood; 2, moderate subarachnoid clots with recognizable arteries; 3, blood clots covering arteries. The total score by adding the scores of all six segments, ranging from 0 to 18, were recorded. Rats with mild SAH (less than 8) at 24 h were excluded from this study.

### 2.6 Short‐term neurological function assessment

At 24 h after SAH, we used the Garcia scoring system and beam balance test to assess the short‐term neurological function. The Garcia scoring system included six parts as spontaneous movement, spontaneous limb movement, forepaw extension, climbing ability, somatosensory and tentacle response. The total score, ranging from 3 to 18, was derived by summing the six test subscores. The rats' capacity to ambulate on a narrow cylindrical wooden beam for a duration of 60 s was assessed through beam balance tests. Scores were recorded as follows: 0, no walking and falling; 1, no walking but remaining on the beam; 2, walking but falling; 3, walking less than 20 cm; 4, walking beyond 20 cm.

### 2.7 Long‐term neurological function assessments

#### 2.7.1 Rotarod test

The rotarod test and Morris water maze were performed to evaluate long‐term neurological function in rats after SAH as previously reported.^7^, ^8^ The rotarod test was performed on days 7, 14, and 21 after SAH to evaluate the abilities of balance and sensorimotor coordination. In the beginning, the rotation speed was five revolutions per minute (RPM) and 10 RPM, respectively, eventually increasing every 5 s by 2 RPM. The time that rats were able to stay on the accelerating rotating cylinder was recorded.

#### 2.7.2 Morris water maze

The water maze test was performed to evaluate learning memory and spatial orientation on days 22–27 after SAH as previously described.^7^, ^8^ On days 22 after SAH, the cued water maze test was conducted. On days 23–26 after SAH, the spatial water maze test was conducted. On days 27, the platform was removed from the water and the probe trail of rats within 60 s was recorded. Video tracking system (Shanghai Xinruan Information Technology Co., Ltd.) was used to record swim distance, escape latency, and probe quadrant duration.

### 2.8 Construction of the pressurizing device

A pressurizing device was developed to simulate the state of intracranial hypertension. The device was a cylindrical structure and sealed with a lid that had an air inlet and an air outlet (Figure 6A). When gas (95% air and 5% CO_2_) entered the device through the inlet, the pressure inside the device was increased. The only difference between the pressurizing device and a regular incubator was the applied pressure. To establish intracranial hypertension model, neurons were incubated in the device, and the pressure level was predetermined as the starting point for the experiment. The pressure value inside the device was recorded every 2 h to ensure a stable pressure.

### 2.9 Isolation and culture of primary hippocampal neurons

Primary hippocampal neurons were obtained from the hippocampus of Sprague–Dawley rats during embryonic days 18–19, following established protocols as described in prior studies.^7^, ^25^, ^26^ In short, the hippocampus was dissected in ice‐cold HBSS, followed by incubation in HBSS supplemented with 2 mg/mL papain for 15 min. In the final stage of digestion, the DNase was added to separate the hippocampal cells in a further way. Then, we seeded them on Poly‐Lysine pretreated culture dishes with neurobasal medium supplemented with 2% B27 (Guangzhou huayun biotech Co., LTD.), 2 mM L‐glutamine and antibiotics. The hippocampal neurons were cultured for 7–14 days and harvested for subsequent experiments.

### 2.10 Western blot

Western blot was performed as described previously.^7^, ^8^ Briefly, the samples and primary neurons protein were extracted with RIPA lysis buffer (Beyotime). Then, a bicinchoninic acid assay kit (Beyotime) was used to determine the protein concentrations. Protein samples were separated by 6%–10% SDS‐PAGE and transferred to polyvinylidene fluoride membranes (Millipore). The membranes were blocked with 5% skim milk for 2 h and incubated overnight at 4°C with the following primary antibodies: Piezo1 (1:500, Cat#15939‐1‐AP, Proteintech), phospho‐LATS1 (1:1000, Cat#8654, Cell Signaling Technology), LATS1 (1:1000, Cat#3477, Cell Signaling Technology), phospho‐YAP (1:1000, Cat#4911, Cell Signaling Technology), YAP (1:1000, Cat#14074, Cell Signaling Technology), Bcl‐2 (1:500, Cat#sc‐7382, Santa Cruz Biotechnology), Bax (1:2000, Cat#50599‐2‐lg, Proteintech), Cleaved Caspase 3 (1:1000, Cat#9662, Cell Signaling Technology), β‐actin (1:20000, Cat#66009‐1‐lg, Proteintech). Chemiluminescence was used to develop the immunoblots after incubated by secondary antibodies that corresponded to the primary antibody used. The densities of immunoblot bands were analyzed using ImageJ software.

### 2.11 Immunofluorescence staining

First, the slides or primary neurons were washed with 0.01 M PBS three times for 5 min after fixation with 4% paraformaldehyde, and then incubated in 0.3% Triton X‐100 for 15 min at room temperature. Slides or neurons were blocked with 5% donkey serum for 2 h at room temperature and incubated with primary antibodies as follows: NeuN (1:200, Cat#ab104224, abcam), Iba1(1:200, Cat#ab5076, abcam), GFAP (1:200, Cat#ab4674, abcam), Piezo1 (1:200, Cat#15939‐1‐AP, Proteintech). The sections or primary neurons were incubated with the secondary antibody that corresponded to the primary antibody used and examined under fluorescence microscope (Olympus Inc). The quantification of fluorescence intensity in the acquired images was performed using the ImageJ software.

### 2.12 Calcium imaging

Calcium imaging of hippocampal neurons was performed using the kit, fluo‐4 am (Beyotime), according to the manufacturer's instructions. Briefly, primary neurons were washed three times with PBS and incubated with working solution fluo‐4 am for 30 min. Then, primary neurons were washed three times to remove fluo‐4 am and incubated for another 30 min. Finally, the calcium imaging was detected by fluorescence microscope. The quantification of fluorescence intensity in the acquired images was performed using the ImageJ software.

### 2.13 Cell viability assay

Neurons were treated with 10 μm oxyhemoglobin (OxyHb) or 12 kpa hydrostatic pressure or both together for 24 h, and the viability of neurons was detected by the cell counting kit‐8 (CCK‐8, Dojindo). Then, neurons were incubated for additional 2 h. The optical density value at 450 nm was detected using a microplate reader.

### 2.14 Generation of neurons with gene knockdown

Lentiviruses for Piezo1 knockdown were purchased from Beijing Tsingke Biotech Co., Ltd. Lentiviral particles were transduced into primary neurons according to the manufacturer's instructions. Western blot and quantitative real‐time PCR were performed to evaluate the knockdown efficiency of the target gene. The shRNA sequence was listed as follows: shPiezo1, 5′‐ GCTTCTACCTGCTGCTCTTTG‐3′.

### 2.15 Quantitative real‐time PCR

Total RNA was obtained from primary neurons using the RNAeasy™ Animal RNA Isolation Kit with Spin Column (Beyotime) as directed by the manufacturer. The gene expression results were normalized to GAPDH. The method of 2^一△△CT^ was used to compare the gene differential expression between the treated groups and the control group. We obtained specific primers from Primer Premier and verified primer specificity via NCBI Primer Blast. The sequences of the primer pairs used were: Piezo1, (forward) 5′‐GACGCCTCACAAGGAAAGC‐3′, (reverse) 5′‐ GCCAGTAGCACGATGACCAG‐3′; GAPDH, (forward) 5′‐GGGCGGTACAACTCAGGTTC‐3′, (reverse) 5′‐CTATGAGACGAGGCTGCTGA‐3′. PrimeScript™ RT Master Mix (Takara) and SYBR Green Master Mix (Vazyme) were used for reverse transcription and amplification, respectively.

### 2.16 TUNEL

To quantify neuronal apoptosis, double staining of the neuron marker NeuN and terminal deoxynucleotidyl transferase dUTP nick end labeling (TUNEL) staining was conducted utilizing the in situ Apoptosis Detection Kit (Elabscience, China) in accordance with the manufacturer's guidelines. The number of TUNEL‐positive neurons was counted.

### 2.17 Statistical analysis

The quantitative data were presented as *mean ± SD* and processed using SPSS 21.0 (IBM, NY). The data were assessed using the Shapiro–Wilk test for normality. The homogeneity of variances was examined with the Levene test or Brown–Forsythe test. Two‐tailed *t* test was used for comparisons between two groups. One‐way analysis of variance (ANOVA) followed by Tukey's post hoc test was used for multiple comparisons among groups. Two‐way ANOVA was applied to analyze the data from long‐term neurobehavioral results. The Mann–Whitney test was used for data that did not exhibit normal distribution. A value of *p* < 0.05 was considered statistically significant.

## 3 RESULTS

### 3.1 Mortality and SAH severity

Of the total 226 rats used, 179 were subjected into SAH induction, of which 35 (19.55%) rats died after SAH. In the group of Sham, all of rats survived. A total of 15 rats were excluded from the study due to mild SAH grade (Figures S1A). Based on the SAH grade outcome, a notable disparity was observed between Sham and SAH rats. However, no statistically significant distinction was found among all SAH groups following SAH induction (Figures S1B). The representative anatomy image of SAH induction was depicted in the Figures S1C.

### 3.2 Temporal expression of Piezo1 in the brain after SAH

**FIGURE 2:**
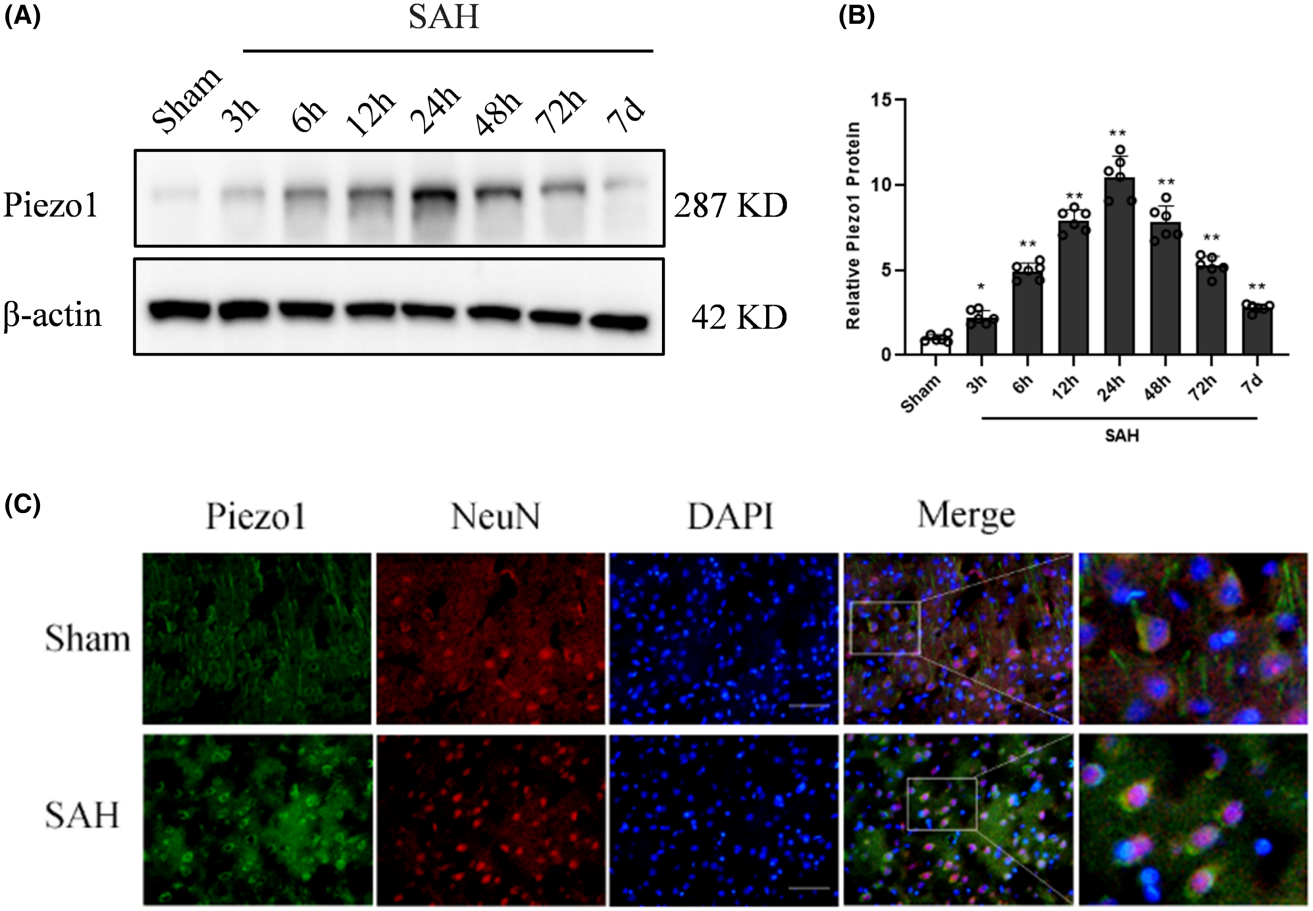
Temporal expression of Piezo1 in the brain after SAH. (A,B) Representative Western blot image and quantitative analysis of Piezo1 from the ipsilateral hemisphere in time course experiment after SAH. Data represent with mean ± SD (*n* = 6). *p < 0.05, **p < 0.01 versus Sham group. (C) Double immunofluorescence staining showed that Piezo1 (green) co‐localized with NeuN‐positive neurons (red) in both Sham and SAH groups (*n* = 4). Scale bar, 50 μm.

The Western blot analysis revealed a significant upregulation of Piezo1 expression following SAH induction, with the highest level observed at 24 h post‐induction, as compared to the Sham group (Figure 2A,B). The results of double immunofluorescence staining revealed that Piezo1 expression was predominantly observed in neurons within the brain. Furthermore, the SAH group exhibited a higher quantity of Piezo1‐positive neurons in comparison to the Sham group (Figure 2C). In addition, we also found that Piezo1 rarely colocalized with the microglia and the astrocyte in our SAH model (Figure S2). Thus, the effect of Piezo1 on neurons was investigated in the subsequent studies.

### 3.3 Piezo1 inhibitor GsMTx4 improved short‐term neurological functions after SAH

**FIGURE 3:**
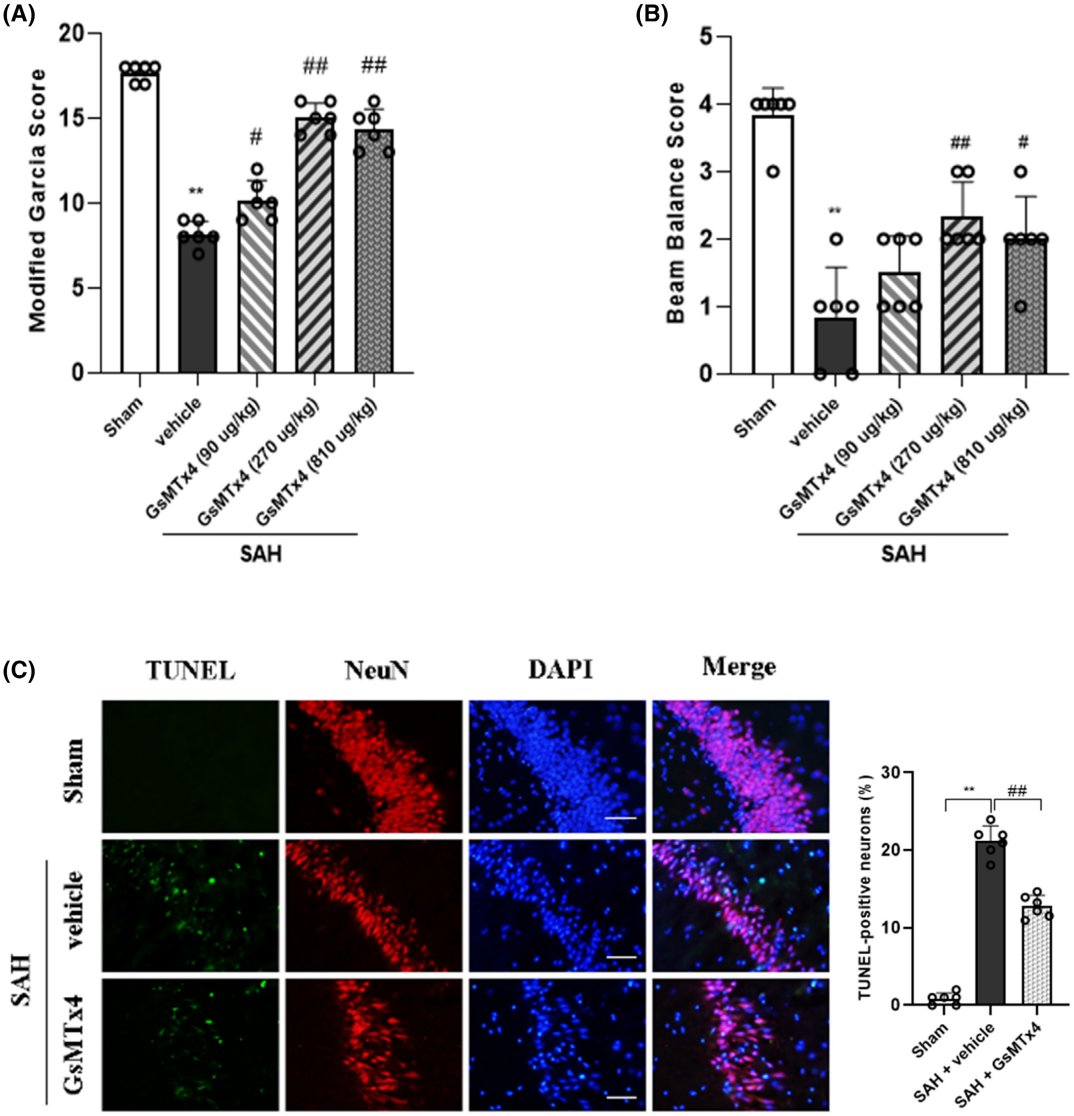
Effects of GsMTx4 on short‐term neurological function and neuronal apoptosis after SAH. Neurological scores assessed with (A) modified Garcia scale and (B) beam balance test at 24 h after SAH. Data represent with mean ± SD (*n* = 6). (C) Representative microphotographs and quantitative analysis of TUNEL‐positive neurons at 24 h after SAH. Data represent with mean ± SD (*n* = 6). Scale bar, 50 μm. **p < 0.01 versus Sham group; ^#^ *p* < 0.05, ^##^ *p* < 0.01 versus SAH + vehicle group.

The short‐term neurobehavioral outcomes were evaluated at 24 h after SAH. Compared to the Sham group, significant neurological deficits were observed in the SAH + vehicle group by modified Garcia score and beam balance test. The Piezo1 inhibitor GsMTx4 at middle dosage (270 μg/kg) and high dosage (810 μg/kg) improved short‐term neurological functions significantly (Figure 3A,B). However, improvement in beam balance test could not be observed in the low dosage (90 μg/kg) group (Figure 3B). GsMTx4 at 270 μg/kg was the most effective dosage and chosen for the following studies.

As SAH leads to neuronal apoptosis, TUNEL staining was utilized to assess the potential neuroprotective effects of GsMTx4. The number of TUNEL‐positive neurons in the SAH + vehicle group significantly increased compared to the Sham group at 24 h post‐SAH; however, treatment with GsMTx4 resulted in a reduction in the number of TUNEL‐positive neurons (Figure 3C).

### 3.4 Piezo1 inhibitor GsMTx4 improved long‐term neurological functions after SAH

**FIGURE 4:**
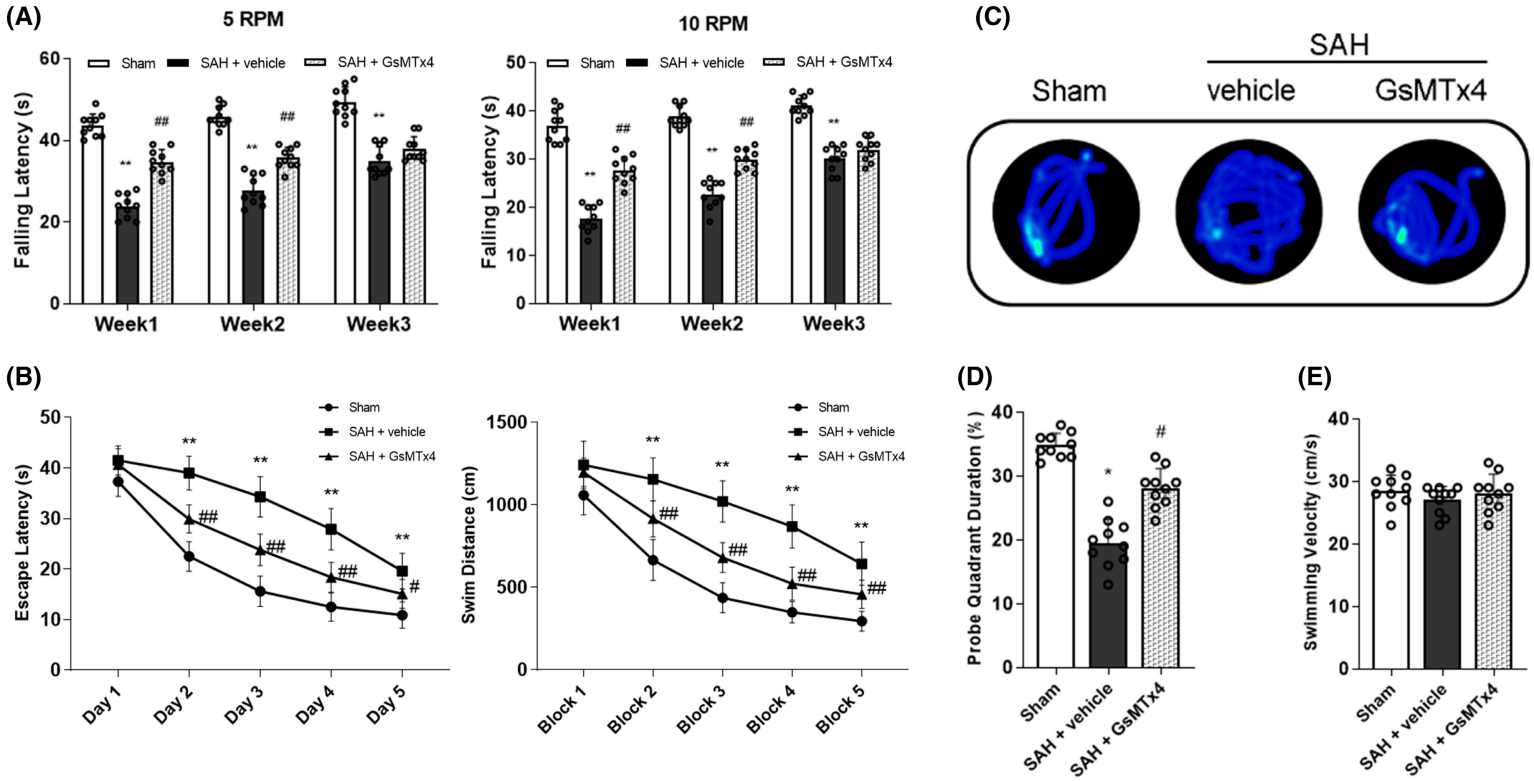
Effects of GsMTx4 on long‐term neurological function after SAH. (A) Rotarod tests of 5 RPM and 10 RPM. (B) Escape latency and swim distance of Morris Water Maze. (C) Representative heatmaps of the probe trial. (D) Quantification of the probe quadrant duration in the probe trial. (E) Swimming velocities of different groups in probe trial. Data represent with mean ± SD (*n* = 10). *p < 0.05, **p < 0.01 versus Sham group; ^#^ *p* < 0.05, ^##^ *p* < 0.01 versus SAH + vehicle group.

Rats had neurological deficits in sensorimotor and memory function after induction of SAH. Therefore, the rotarod test and Morris water maze were employed to evaluate the enduring neurological capabilities following SAH. In the rotarod test, the SAH + vehicle group exhibited a significantly diminished falling latency during both 5 RPM and 10 RPM accelerating velocity assessments in comparison to the Sham group. However, GsMTx4 administration ameliorated the rotarod outcome of SAH rats at both 5 RPM and 10 RPM accelerating velocity tests compared to the SAH + vehicle group in the weeks 1 and 2 (Figure 4A).

In the Morris water maze, we found that rats in the SAH + vehicle group spent more time to find the platform submerged in the water and had a longer swim distance than rats in the Sham group (Figure 4B,C). However, escape latency and swim distance were significantly decreased by GsMTx4 administration (Figure 4B,C). We also found that rats in the SAH + vehicle group spent less time in the target quadrant than rats in the Sham group, and GsMTx4 increased the time rats spent in the probe quadrant (Figure 4D). The swimming velocity was not significantly different among all the groups (Figure 4E).

### 3.5 Piezo1 selective agonist Yoda1 or the Hippo pathway agonist MY‐875 reversed the anti‐apoptotic effects of Piezo1 knockdown

**FIGURE 5:**
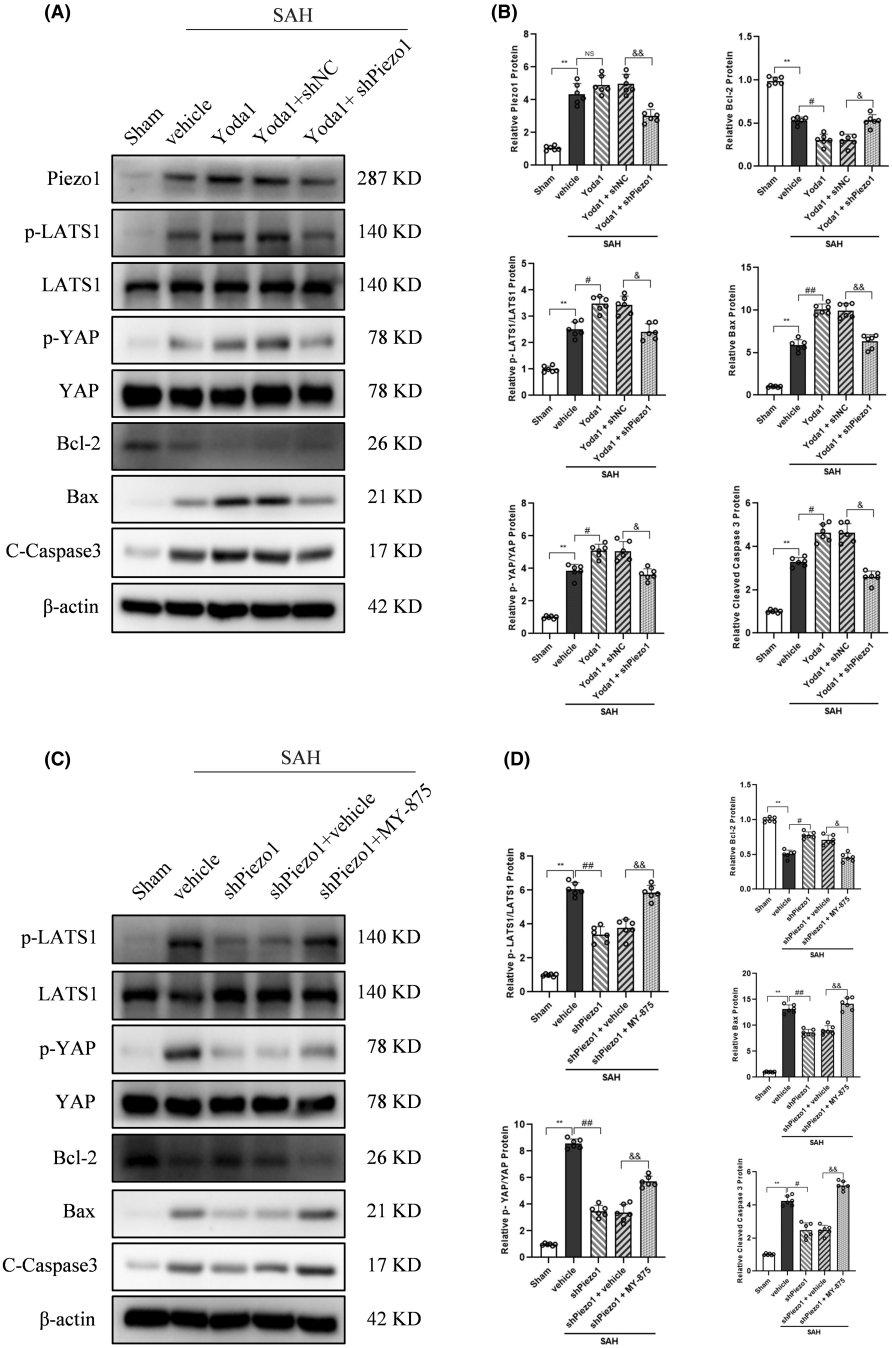
Piezo1 selective agonist Yoda1 or the Hippo pathway agonist MY‐875 reversed the anti‐apoptotic effects of Piezo1 knockdown at 24 h after SAH. (A,B) Representative Western blot images and quantitative analysis of Piezo1, p‐LATS1, p‐YAP, Bcl‐2, Bax, and Cleaved Caspase 3. Data represent with mean ± SD (*n* = 6). **p < 0.01 versus Sham group; ^#^ *p* < 0.05, ^##^ *p* < 0.01 versus SAH + vehicle group; ^&^ *p* < 0.05, ^&&^ *p* < 0.01 versus SAH + Yoda1 + shNC group; NS, no significance. (C,D) Representative Western blot images and quantitative analysis of p‐LATS1, p‐YAP, Bcl‐2, Bax, and Cleaved Caspase 3. Data represent with mean ± SD (*n* = 6). **p < 0.01 versus Sham group; ^#^ *p* < 0.05, ^##^ *p* < 0.01 versus SAH + vehicle group; ^&^ *p* < 0.05, ^&&^ *p* < 0.01 versus SAH + shPiezo1 + vehicle group.

The Piezo1 agonist Yoda1 and short hairpin RNA for Piezo1 knockdown were utilized to investigate their effects on neuronal apoptosis in vivo. The Western blot analysis showed that Bax and cleaved caspase 3 expression were significantly increased, and Bcl‐2 expression was decreased in the group of SAH + vehicle, compared to the Sham group (Figure 5A,B). Yoda1 further aggravated SAH‐induced apoptosis. Furthermore, the treatment of Piezo1 knockdown attenuated neuronal apoptosis caused by Yoda1 in rats after SAH (Figure 5A,B). The expression of p‐LATS1 and p‐YAP were significantly increased in the group of SAH + vehicle, compared to the Sham group (Figure 5A,B). Yoda1 administration further activated the Hippo pathway. Furthermore, the treatment of Piezo1 knockdown diminished the effects on Hippo pathway caused by Yoda1 in rats after SAH (Figure 5A,B).

In order to confirm the Hippo pathway as the downstream signaling pathway of Piezo1 activation induced by SAH, the Hippo pathway agonist MY‐875 was used. The results of the Western blot indicated that Piezo1 knockdown resulted in anti‐apoptotic effects in the SAH + shPiezo1 group, compared to the SAH + vehicle group (Figure 5C,D). Additionally, the introduction of the Hippo pathway agonist MY‐875 reversed the neuroprotective effects of Piezo1 knockdown in the SAH + shPiezo1 + MY‐875 group, as compared to the SAH + shPiezo1 + vehicle group (Figure 5C,D).

### 3.6 Intracranial hypertension mimicked by the pressurizing device induced Piezo1 expression in primary neurons

**FIGURE 6:**
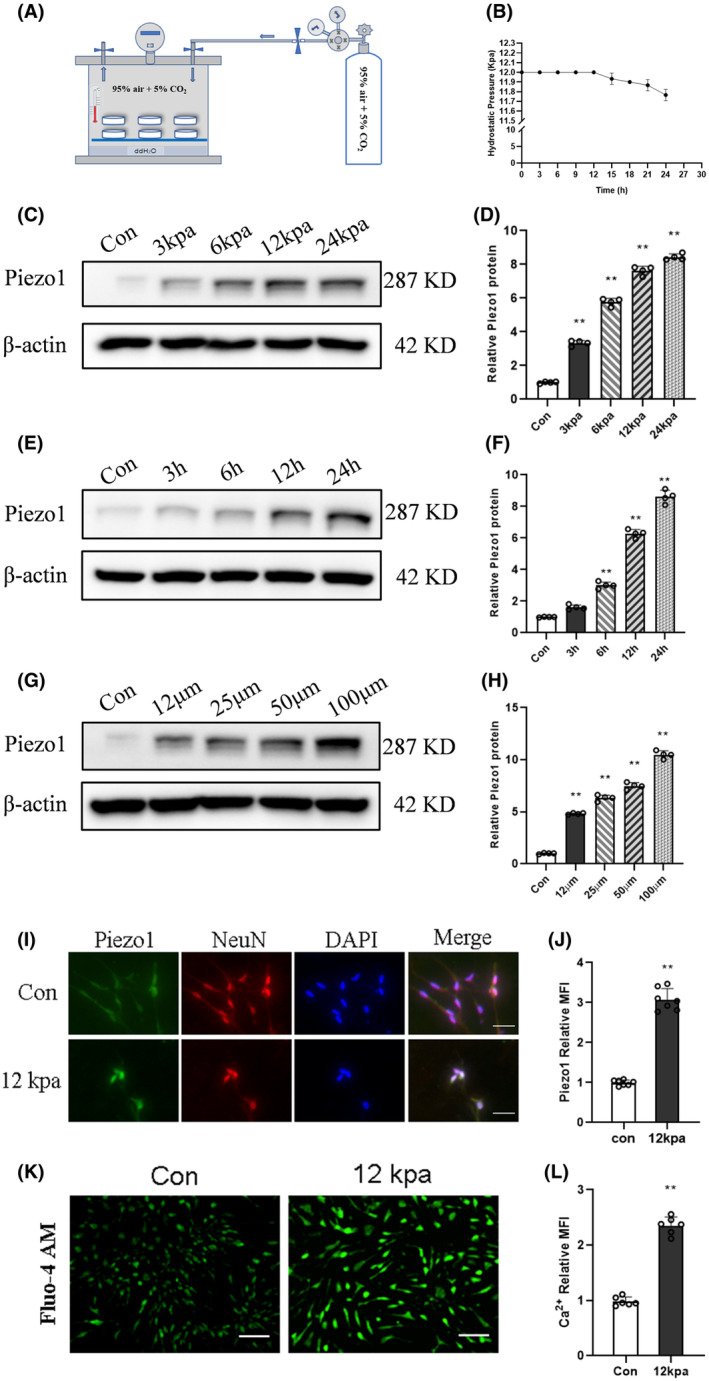
Intracranial hypertension mimicked by the pressurizing device induced Piezo1 expression in primary neurons. (A) Diagram of the pressurizing device. (B) Pressure values of the pressurizing device within 24 h. (C,D) Representative immunoblots and corresponding densitometry analysis of Piezo1 in primary neurons treated with different pressures for 24 h. Data are shown as mean ± SD (*n* = 4). (E,F) Representative immunoblots and corresponding densitometry analysis of Piezo1 in primary neurons treated with 12 kpa for different times. Data are shown as mean ± SD (*n* = 4). (G,H) Representative immunoblots and corresponding densitometry analysis of Piezo1 in primary neurons treated with different concentrations of OxyHb for 24 h. Data are shown as mean ± SD (*n* = 4). (I,J) Representative fluorescence images and summarized data showing the change of Piezo1 in primary neurons treated with 12 kpa for 24 h. Data are shown as mean ± SD (*n* = 6). Scale bar, 50 μm. (K,L) Fluo‐4 AM was added to primary neurons treated with 12 kpa for 24 h, and the fluorescence signals of calcium were detected. Data are shown as mean ± SD (*n* = 6). Scale bar, 100 μm. **p < 0.01 versus Con group.

A pressurizing device was developed to simulate intracranial hypertension in SAH (Figure 6A). Previous research indicated that rats experience a peak intracranial pressure of approximately 12 kpa after SAH.^27^ To establish a stable hypertensive environment, the airtight function of the pressurizing device was initially assessed (Figure 6B).

Immunofluorescence staining was conducted to identify NeuN‐positive cells, revealing that the proportion of NeuN‐positive cells within the isolated neurons exceeded 95%; the result indicated the presence of highly pure primary neurons suitable for subsequent experimental investigations (Figure 6I). Then, neurons were incubated in the pressurizing device and the expression of Piezo1 was detected following. We found that Piezo1 expression was upregulated in abundance with elevation of pressure and was increased over time (Figure 6C–F). In addition, our results demonstrated that OxyHb activated Piezo1 in a concentration‐dependent manner in vitro (Figure 6G,H). Immunofluorescence staining and calcium imaging were employed to elucidate the Piezo1 activation caused by hypertension. The expression of Piezo1 and the influx of calcium in primary neurons were significantly increased in the hypertensive environment of 12 kpa, as compared to the control group (Figure 6I–L).

### 3.7 Piezo1 induced by intracranial hypertension promoted neuronal apoptosis

**FIGURE 7:**
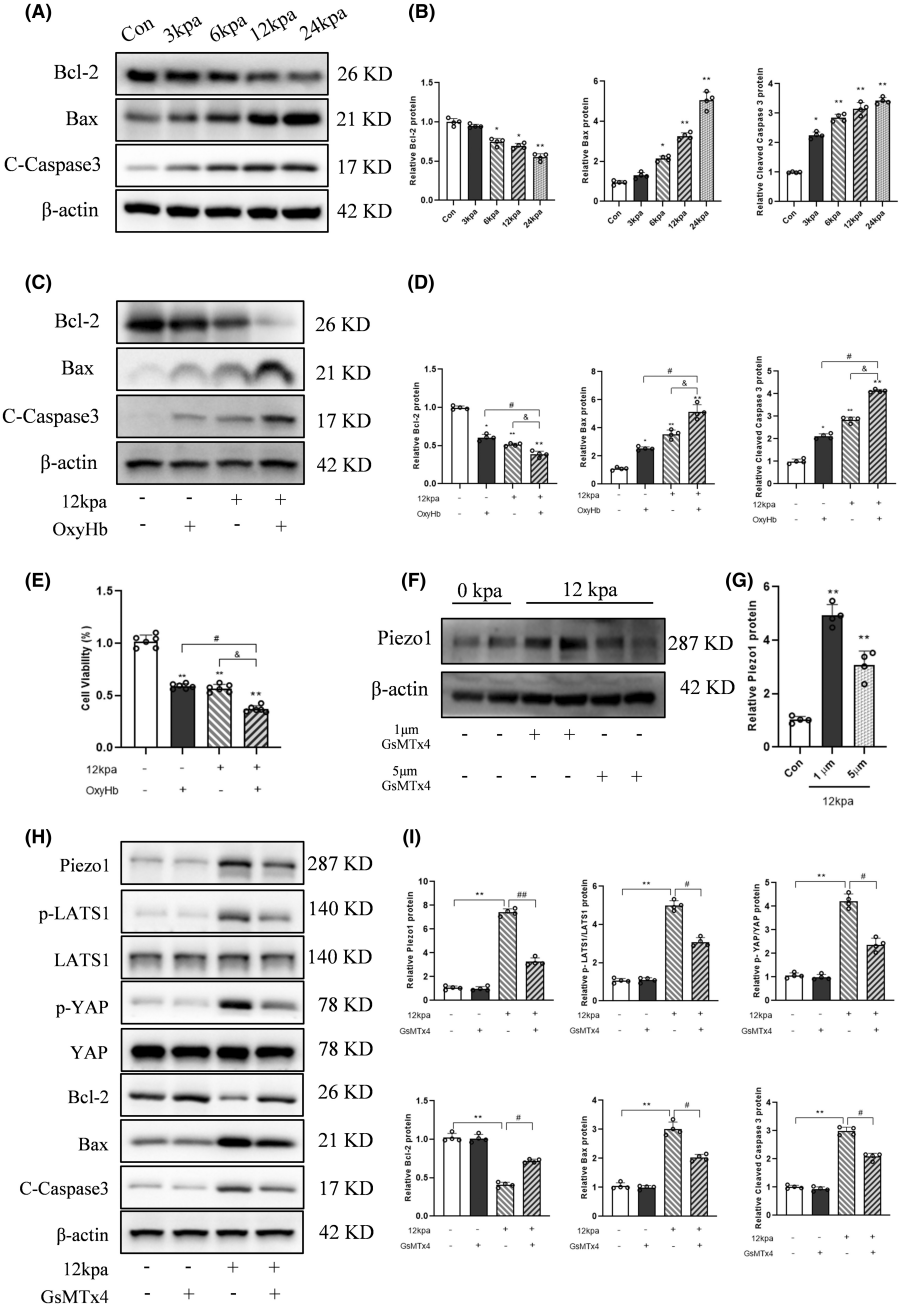
Piezo1 expression induced by intracranial hypertension promoted neuronal apoptosis, which was attenuated with GsMTx4 treatment. (A,B) Representative immunoblots and corresponding densitometry analysis of Bcl‐2, Bax, and Cleaved Caspase 3 in primary neurons treated with different pressures for 24 h. Data are shown as mean ± SD (*n* = 4). *p < 0.05, **p < 0.01 versus Con group. (C,D) Representative immunoblots and corresponding densitometry analysis of Bcl‐2, Bax, and Cleaved Caspase 3 in primary neurons treated with 10 μm OxyHb or 12 kpa or both together for 24 h. Data are shown as mean ± SD (*n* = 4). *p < 0.05, **p < 0.01 versus Con group; ^#^ *p* < 0.05 versus OxyHb group; ^&^ *p* < 0.05 versus 12 kpa group. (E) Primary neurons treated with 10 μm OxyHb or 12 kpa or both together for 24 h, viability of neurons detected by CCK‐8. Data are shown as mean ± SD (*n* = 6). **p < 0.01 versus Con group; ^#^ *p* < 0.05 versus OxyHb group; ^&^ *p* < 0.05 versus 12 kpa group. (F,G) Representative immunoblots and corresponding densitometry analysis of Piezo1 in primary neurons pretreated with 1 μm or 5 μm GsMTx4 followed by 12 kpa for 24 h. Data are shown as mean ± SD (*n* = 4). **p < 0.01 versus Con group. (H,I) Representative immunoblots and corresponding densitometry analysis of Piezo1, p‐LATS1, p‐YAP, Bcl‐2, Bax, and Cleaved Caspase 3 in primary neurons pretreated with 5 μm GsMTx4 followed by 12 kpa for 24 h. Data are shown as mean ± SD (*n* = 4). **p < 0.01 versus Con group; ^#^ *p* < 0.05, ^##^ *p* < 0.01 versus 12 kpa + GsMTx4 group.

Primary neurons were subjected to different pressures for 24 h, and apoptosis‐related proteins were detected. We found that Bax and cleaved caspase 3 were significantly increased with elevated pressure, but a gradual reduction in Bcl‐2 expression (Figure 7A,B). Next, primary neurons were treated with 10 μm OxyHb or 12 kpa hydrostatic pressure or both together for 24 h, and apoptosis‐related proteins were detected. Treatment of 10 μm OxyHb or 12 kpa hydrostatic pressure promoted neuronal apoptosis, and this effect was further enhanced when neurons were treated with OxyHb and 12 kpa together (Figure 7C–E).

Then, GsMTx4 was adopted to inhibit Piezo1 expression in vitro. Intracranial hypertension induced Piezo1 protein expression, which was markedly suppressed by GsMTx4 at 1 and 5 μM (Figure 7F,G). Intracranial hypertension caused increased abundance of Bax and cleaved caspase 3, accompanied with decreased protein expression of Bcl‐2, both of which were partially diminished by Piezo1 inhibitor GsMTx4 (Figure 7H,I). Intracranial hypertension also increased the expression of p‐LATS1 and p‐YAP, which was partially prevented by GsMTx4 (Figure 7H,I). TUNEL staining showed that the number of TUNEL‐positive neurons in the 12 kpa group significantly increased compared to the Con group; however, treatment with GsMTx4 resulted in a reduction in the number of TUNEL‐positive neurons (Figure S3).

### 3.8 Knockdown of Piezo1 protected primary neurons against intracranial hypertension‐induced neuronal apoptosis via inhibiting the Hippo pathway

**FIGURE 8:**
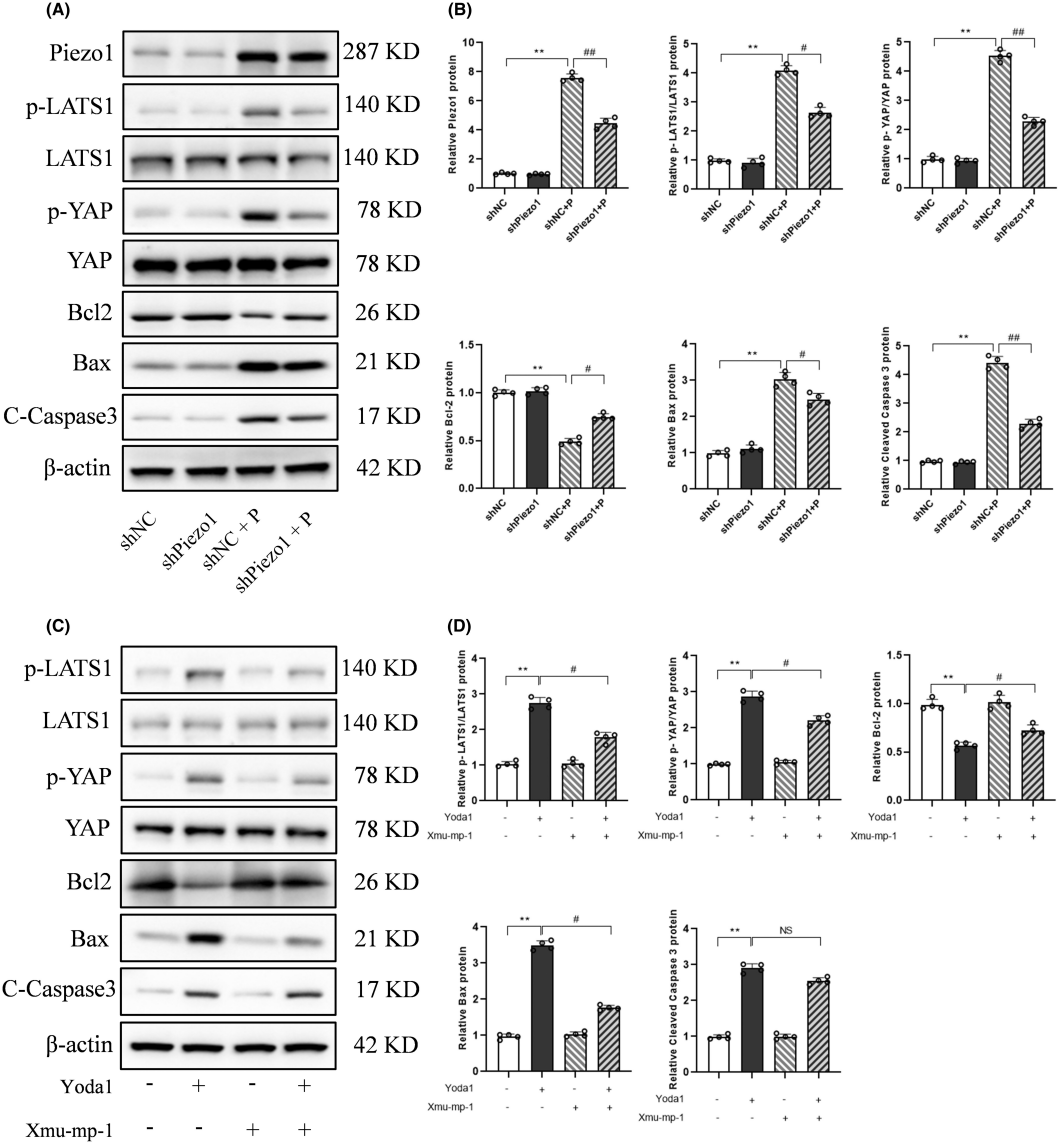
Knockdown of Piezo1 protected primary neurons against intracranial hypertension‐induced neuronal apoptosis via inhibiting the Hippo pathway. (A,B) Representative immunoblots and corresponding densitometry analysis of Piezo1, p‐LATS1, p‐YAP, Bcl‐2, Bax, and Cleaved Caspase 3 in primary neurons transfected with shPiezo1 or shNC followed by 12 kpa treatment for 24 h. Data are shown as mean ± SD (*n* = 4). **p < 0.01 versus shNC group; ^#^ *p* < 0.05, ^##^ *p* < 0.01 versus shPiezo1 + P group. P, 12 kpa hydrostatic pressure. (C,D) Representative immunoblots and corresponding densitometry analysis of p‐LATS1, p‐YAP, Bcl‐2, Bax, and Cleaved Caspase 3 in primary neurons pretreated 50 μm Xmu‐mp‐1 followed by 1 μm Yoda1 for 24 h. Data are shown as mean ± SD (*n* = 4). **p < 0.01 versus Con group; ^#^ *p* < 0.05 versus Yoda1 + Xmu‐mp‐1 group; NS, no significance.

GsMTx4 was not a specific Piezo1 antagonist, so lentiviral transduction of shRNA was introduced to elucidate the function of Piezo1 in neurons in respond to intracranial hypertension. The lentiviral‐mediated knockdown efficiency of Piezo1 was verified at both the mRNA and protein levels (Figure S4A–C). Due to superior knockdown efficiency of shPiezo1‐3, it was chosen for subsequent experiments. Piezo1 knockdown attenuated intracranial hypertension‐induced increased protein of Bax and cleaved caspase 3, and reversed protein level of Bcl‐2 to control levels (Figure 8A,B). Intracranial hypertension also increased the expression of p‐LATS1 and p‐YAP, which was partially prevented by Piezo1 knockdown (Figure 8A,B).

In order to conduct a more comprehensive examination of the involvement of the Hippo pathway in Piezo1‐induced neuronal apoptosis, the Hippo pathway was suppressed through the utilization of Xmu‐mp‐1. Compared to the Yoda1 group, the protein abundance of Bax was greatly decreased and Bcl‐2 protein expression was markedly upregulated in the Yoda1 + Xmu‐mp‐1 group, indicating a suppression of apoptosis caused by the Hippo pathway inhibition (Figure 8C,D).

## 4 DISCUSSION

In this study, we assessed the anti‐apoptotic effects of Piezo1 inhibition and explored the underlying mechanism of Piezo1 inhibition in rats after SAH. Then, we verified the effect of Piezo1 activation on neuronal apoptosis and the Hippo pathway activation in respond to intracranial hypertension in vitro. The main novel findings of this study are: (1) Piezo1 inhibition markedly reduced neuronal apoptosis after SAH, which was accompanied by the improvement of neurological deficits. (2) Intracranial hypertension mimicked by the pressurizing device verified the result of hypertension‐induced neuronal apoptosis, which can be reversed via Piezo1 inhibition. (3) The Hippo signaling pathway is involved in Piezo1 activation‐induced neuronal apoptosis in respond to intracranial hypertension. Taken together, our results suggest that Piezo1 activation caused by hypertension induced neuronal apoptosis via activating the Hippo pathway.

In the central nervous system, signals blockage caused by massive neurons loss may lead to neurological deficits after SAH. Therefore, neuronal apoptosis has been regarded as a crucial pathophysiologic mechanism in the process of SAH.^28^, ^29^ According to this rationale, many studies have been made to inhibit neuronal apoptosis and improve neurological function following SAH from the aspects of hemoglobin toxicity, oxidative stress, and inflammation.^7^, ^10^, ^12^, ^30^ However, effects of intracranial hypertension on neurons are rarely explored. The majority of SAH patients experienced the symptom of severe headache caused by intracranial hypertension. In addition, intracranial hypertension is not only a fundamental symptom in SAH, but in other central nervous system diseases.^31^, ^32^ Consequently, comprehending the underlying mechanisms of intracranial hypertension can provide valuable insights into the pathophysiology of these specific diseases.

Piezo1 is a mechanosensitive ion channel protein, which can convert extracellular mechanical stimulus into intracellular electrical signals. Previous studies demonstrated that Piezo1 expression was induced by stiffness, mechanical stretch, or compression.^33^, ^34^, ^35^ But hydrostatic pressure generated by the pressurizing device can better characterize the intracranial pressure in patients with SAH. In this study, we found that intracranial hypertension simulated by the pressurizing device succeeded in increasing Piezo1 expression in a pressure‐dependent and time‐dependent manner when primary neurons were subjected to the hypertensive environment. Besides, we found that OxyHb induced Piezo1 expression in a concentration‐dependent manner, and the similar conclusion was reported in other studies as well. In renal fibrosis, a study reported that TGF‐β1 administration increased Piezo1 expression in proximal tubular cells.^33^ Thus, the pressurizing device was employed solely for the purpose of examining the impact of mechanical stimulus on neurons. In this study, results of calcium imaging and immunofluorescence staining demonstrated that hydrostatic pressure of 12 kpa effectively increased extracellular Ca^2+^ influx and Piezo1 expression, respectively. These results illustrated that intracranial hypertension generated by the pressurizing device promoted Piezo1 expression.

Results from the double immunofluorescence staining revealed that Piezo1 expression was predominantly observed in neurons of rats following SAH. The treatment of Piezo1 inhibition resulted in a significant reduction in neuronal apoptosis in rats after SAH, concomitant with improvements in neurological deficits. In our in vitro study, apoptosis‐associated protein Bax and cleaved caspase 3 were significantly increased and Bcl‐2 was decreased when primary neurons subjected to 12 kpa intracranial hypertension environment. Consistent with our results, a significantly decreased viability was proved in degenerated human intervertebral discs; the increased expression of Piezo1 was observed when cells subjected to high‐intensity stress treatment.^35^ Nevertheless, OxyHb administration also induced neuronal apoptosis. The combination of 12 kpa and OxyHb treatment further intensified neuronal apoptosis in primary neurons.

Currently, the in vitro research model for SAH is constrained due to the inadequate representation of the in vivo SAH model by cells treated solely with oxyhemoglobin or hemoglobin. The pathophysiological mechanism associated with SAH is intricate, encompassing factors such as intracranial hypertension, hemoglobin toxicity, hypoxia, energy deficit, and others.^17^, ^36^, ^37^ Except for oxyhemoglobin administration, the factor of hypertension was introduced to neurons cultivation to examine the effects on neuronal apoptosis in this study. An ideal in vitro SAH research model may be developed when the factors of hypoxia and energy deficit are added in the pressurizing device, which will be the focus of our next research.

It is noteworthy to mention that the upregulation of Piezo1 expression has been observed to promote tumor cell proliferation in various carcinoma types.^38^, ^39^, ^40^ However, an exception to this trend existed in non‐small cell lung cancer, where Piezo channels were found to function as tumor suppressors.^41^ The findings appeared to contradict our conclusion that neuronal apoptosis was induced by Piezo1 activation through intracranial hypertension. It is worth noting that carcinoma cells exhibit diminished sensitivity to contact inhibition compared to normal cells,^42^ which could potentially account for the divergent conclusions reached.

The Hippo signaling pathway plays a crucial role in cell proliferation, apoptosis, organ growth, and tissue homeostasis.^23^, ^42^, ^43^ Several studies have shown that the Hippo pathway involved in mechanical stimulus is regulated by Piezo1.^34^, ^39^ Nevertheless, the relationship between Piezo1 and the Hippo pathway in SAH remains elusive. MST1/2, LATS1/2, and YAP are core factors of the Hippo pathway.^42^ Following the Hippo pathway activation, MST1/2, LATS1/2, and YAP are phosphorylated subsequently. The compound MY‐875, acting as a Hippo pathway agonist, promoted cellular apoptosis through upregulation of phosphorylated LATS and YAP proteins.^44^ The Hippo pathway antagonist Xmu‐mp‐1 effectively alleviated the neurological impairments, neuroinflammation and white matter injury in mice after SAH.^45^ In our study, results demonstrated that p‐LATS1 and p‐YAP expression were attenuated with Piezo1 knockdown, and the neuroprotective effects of Piezo1 knockdown were reversed with MY‐875 treatment in rats following SAH. In addition, neuronal apoptosis caused by Yoda1 administration was partially reversed by Xmu‐mp‐1 treatment in vitro.

In this study, there are several points that merit further discussion. First, the Piezo1 inhibitor GsMTx4 is not selective, so we cannot exclude the possibility that other factors are involved. Second, a previous study reported that Piezo1 activation inhibited Hippo pathway through regulating MAPK signaling pathway in hepatocellular carcinoma,^40^ so it is possible that other pathways may participate in Piezo1‐induced neuronal apoptosis.

In summary, our results demonstrated that Piezo1 inhibition attenuated neuronal apoptosis and improved the outcome of neurological deficits in rats after SAH. Activation of Piezo1 by intracranial hypertension induced neuronal apoptosis via activating the Hippo pathway. Furthermore, this study incorporated the variables of OxyHb and intracranial hypertension into the cultivation of neurons, resulting in a compounded impact on neuronal apoptosis. An optimal in vitro model for studying SAH could potentially be created by incorporating additional factors such as hypoxia and energy deficit into the pressurizing device.

## AUTHOR CONTRIBUTIONS

F.L., J.Z., and Z.F. participated in the experimental design, data analysis and interpretation, and manuscript preparation. Z.Z., Y.W., J.W., and J.D. performed the experiments. Y.W. and L.C. collected and analyzed the data. J.Z. and Z.F. drafted the manuscript and F.L. proofread the language. All authors read and approved the final manuscript.

## FUNDING INFORMATION

This study was supported by the National Natural Science Foundation of China (81870944, F.L.).

## CONFLICT OF INTEREST STATEMENT

The authors declare no conflicts of interest.

## Supporting information


Data S1.

Figures S1‐S4.

## Data Availability

The original contributions presented in this study are included in the article materials, and further inquiries can be directed to the corresponding authors.
