# Supplementary material for: Activation of Piezo1 by intracranial hypertension induced neuronal apoptosis via activating hippo pathway
Source: CNS Neurosci Ther. 2024 Sep 27;30(9):e14872. doi: 10.1111/cns.14872 (PMC11427798; doi:10.1111/cns.14872)

Full unedited blot for Figure 2A

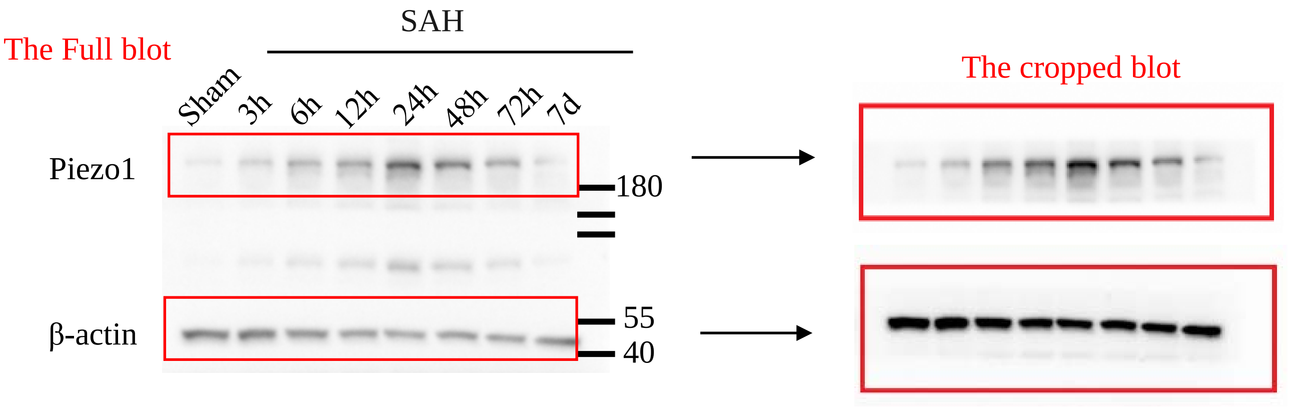

Full unedited blot for Figure 5A

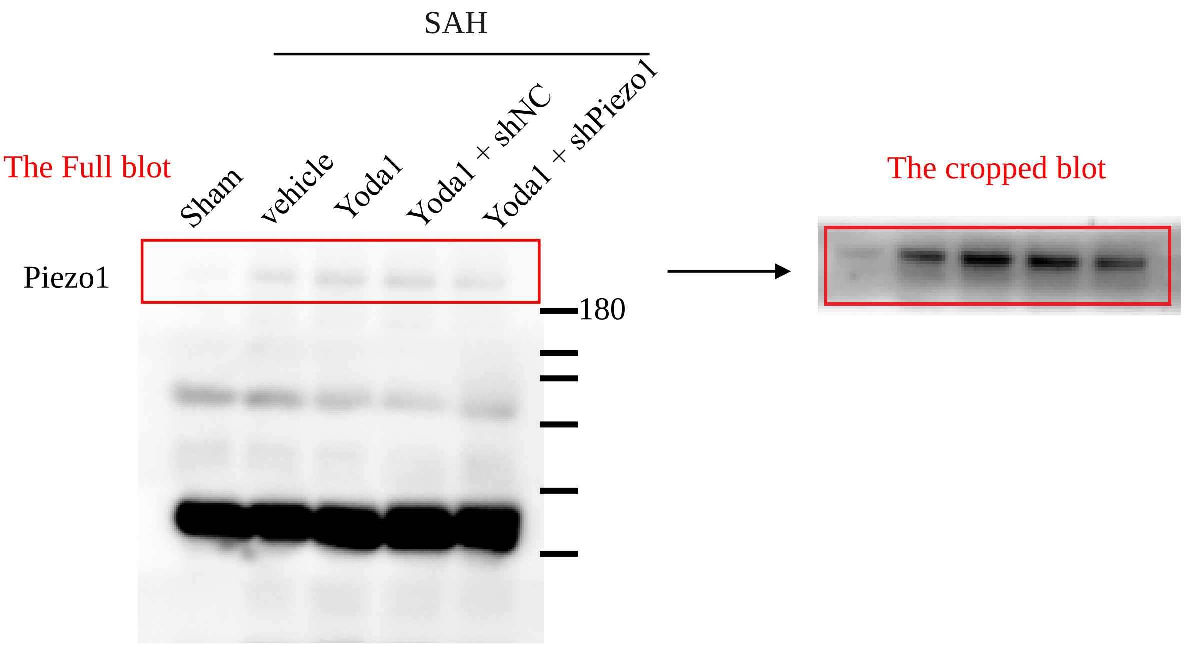

Full unedited blot for Figure 5A

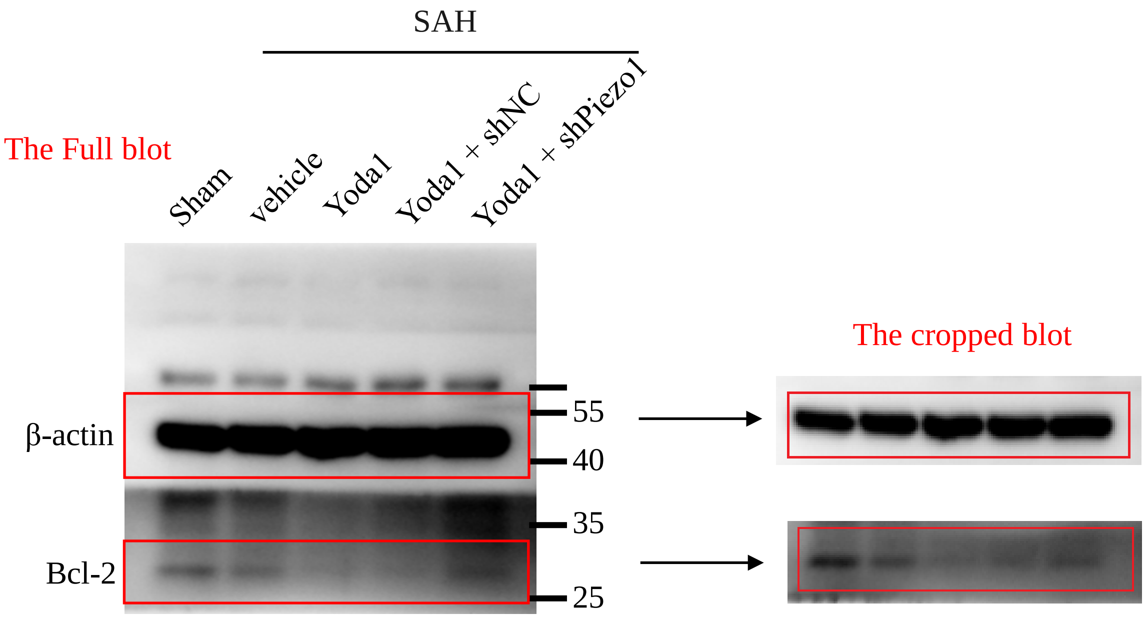

Full unedited blot for Figure 5A

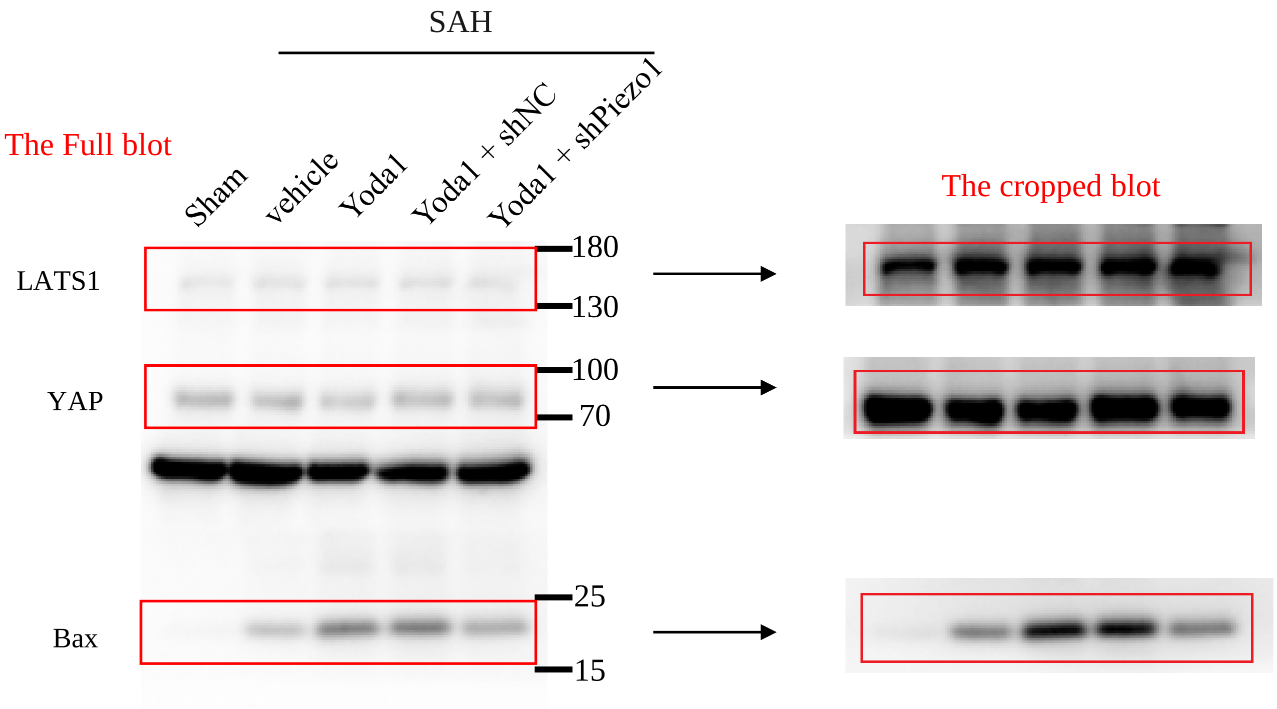

Full unedited blot for Figure 5A

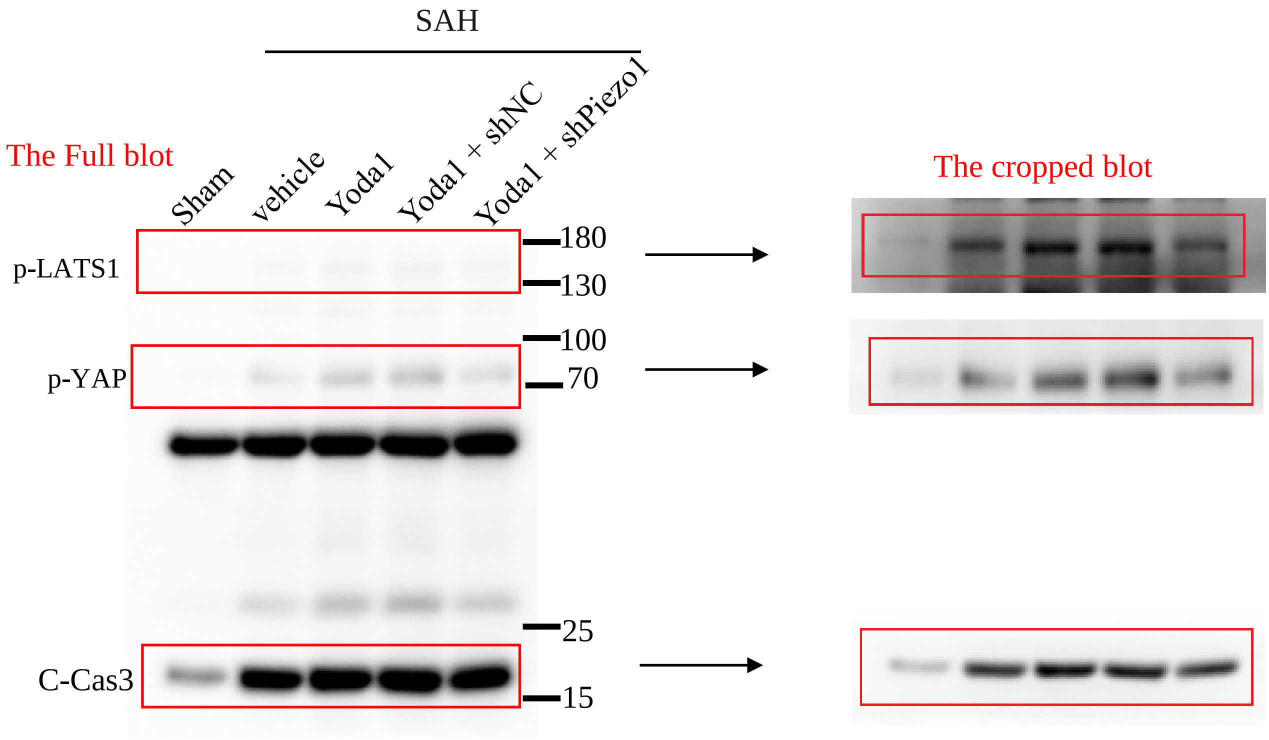

Full unedited blot for Figure 5C

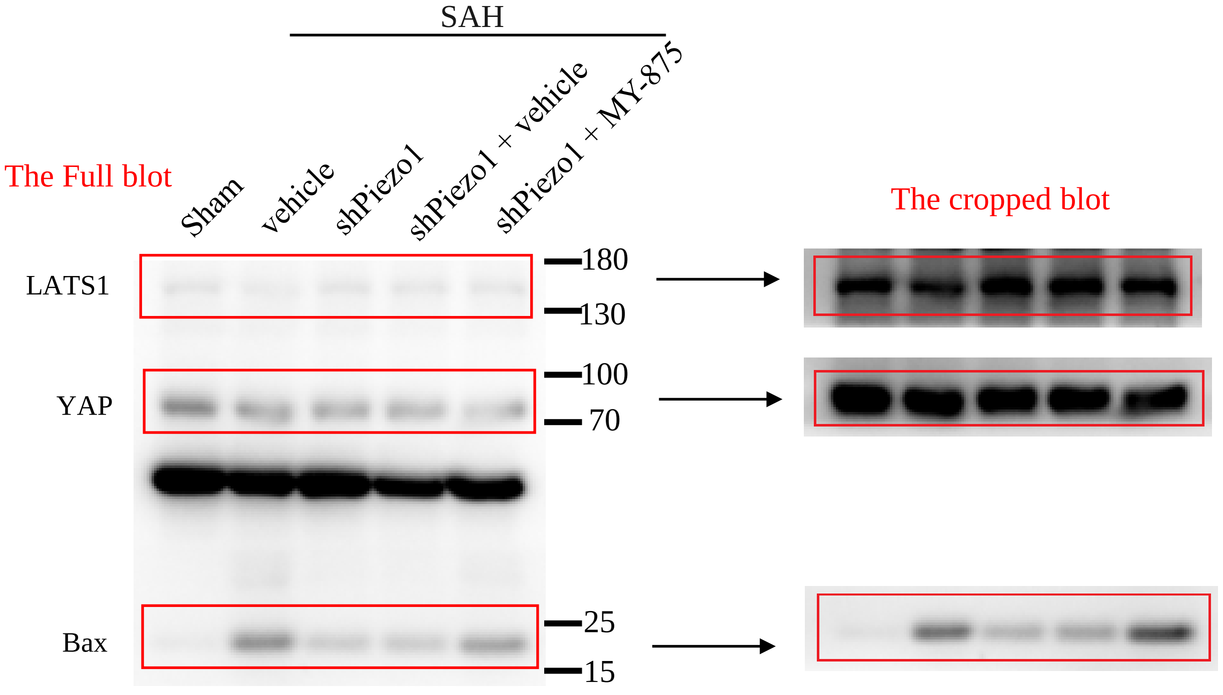

Full unedited blot for Figure 5C

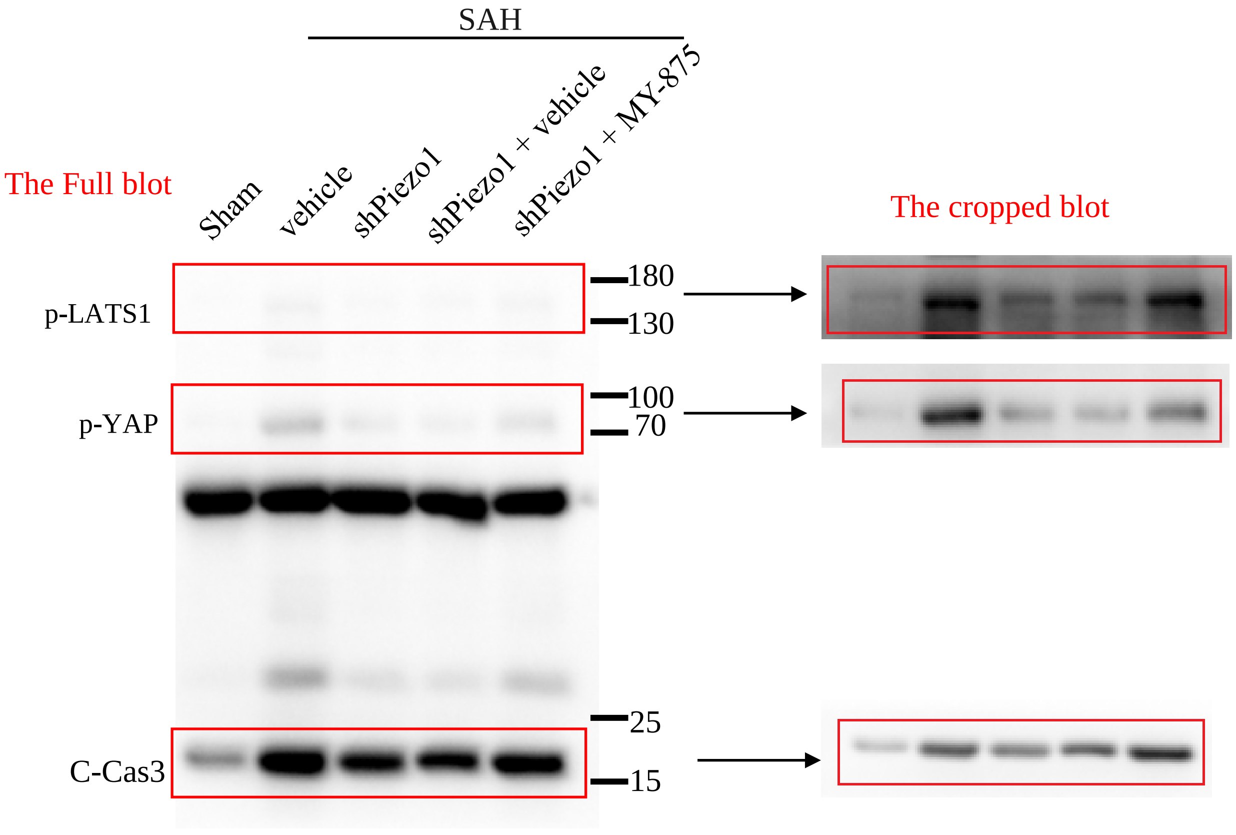

Full unedited blot for Figure 5C

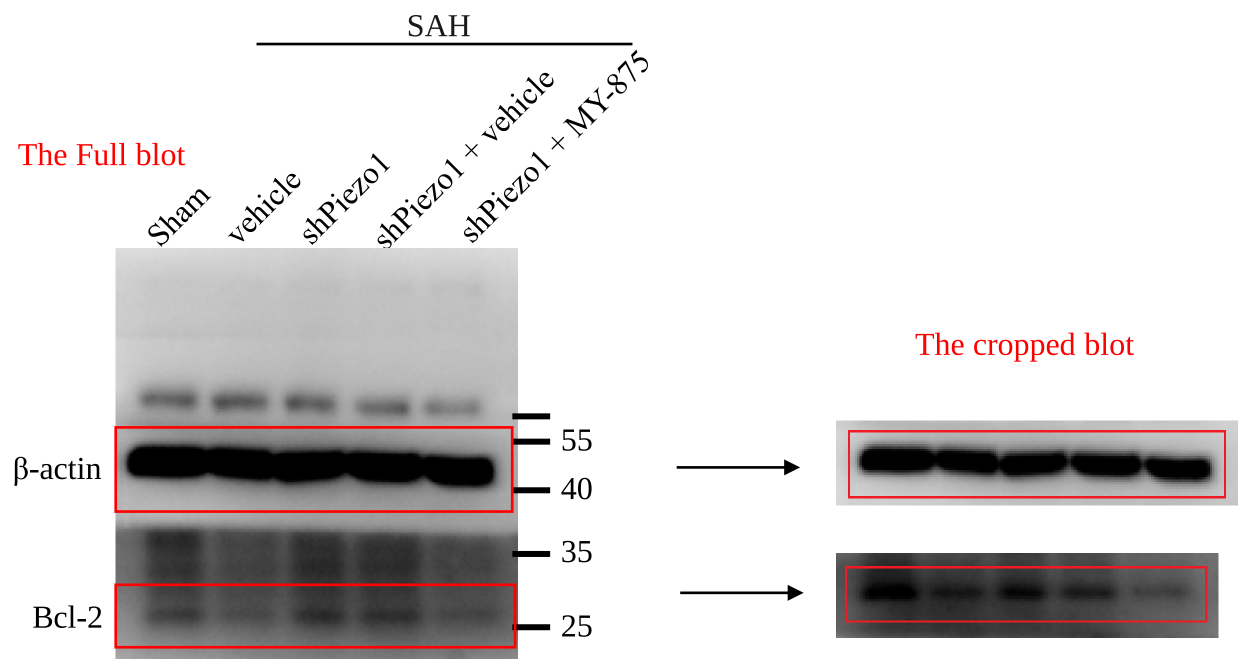

Full unedited blot for Figure 6C

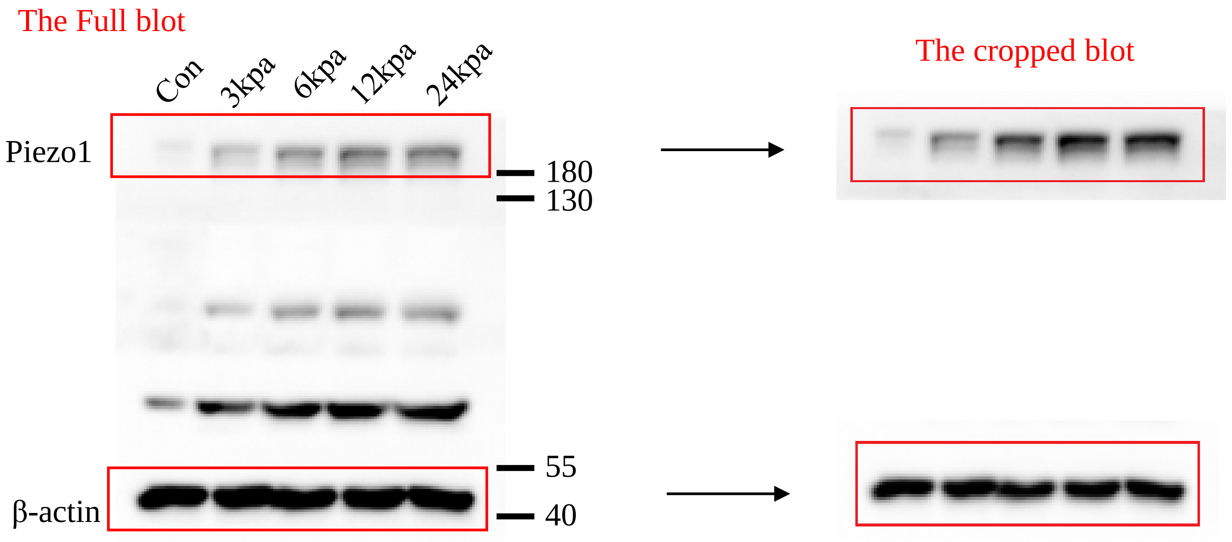

Full unedited blot for Figure 6E

The Full blot

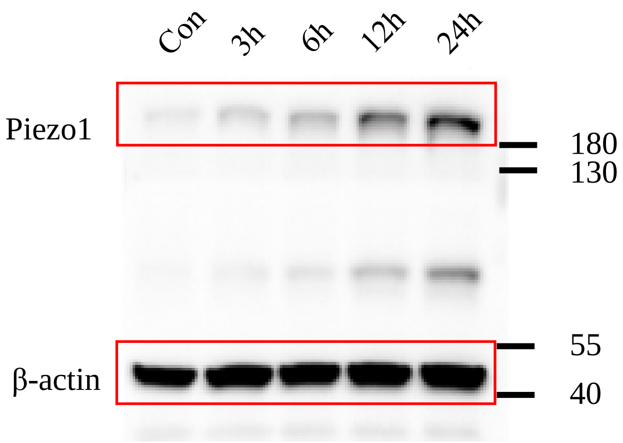

The cropped blot

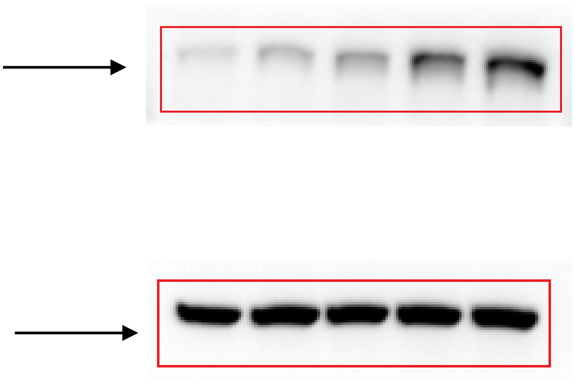

Full unedited blot for Figure 6G

The Full blot

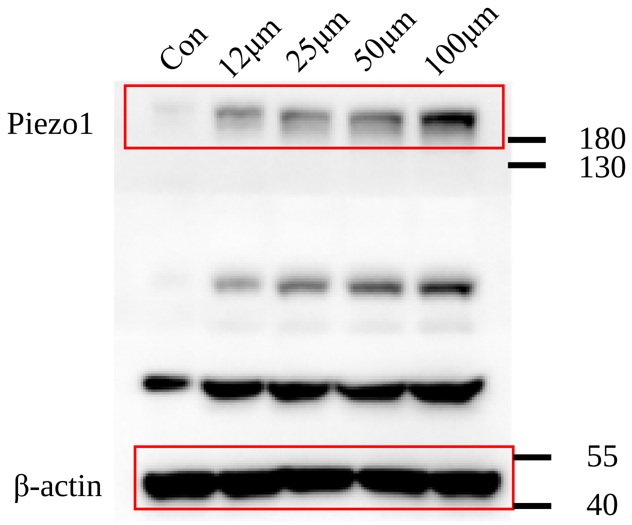

The cropped blot

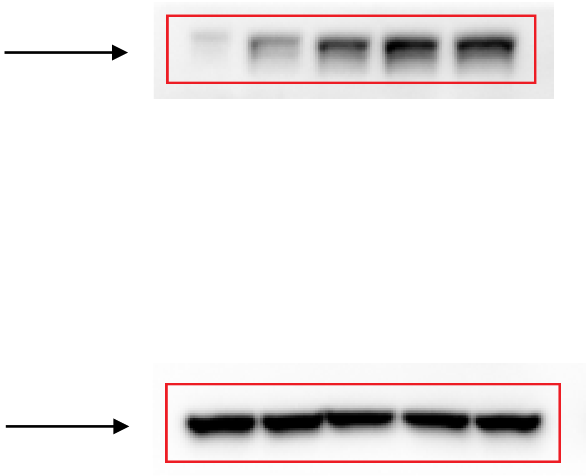

Full unedited blot for Figure 7A

The Full blot

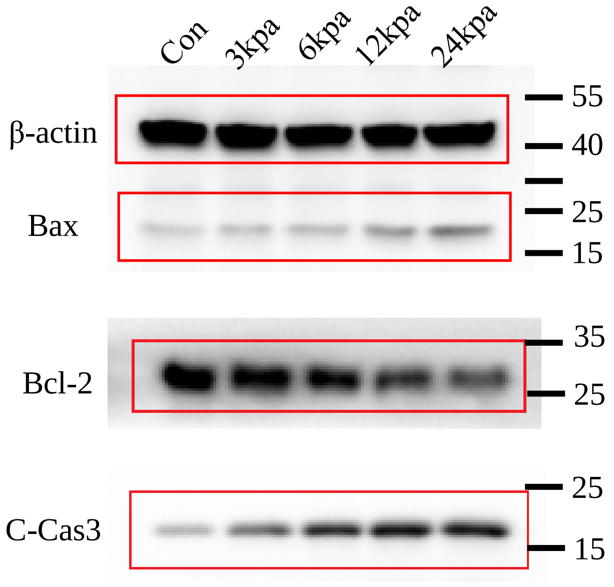

The cropped blot

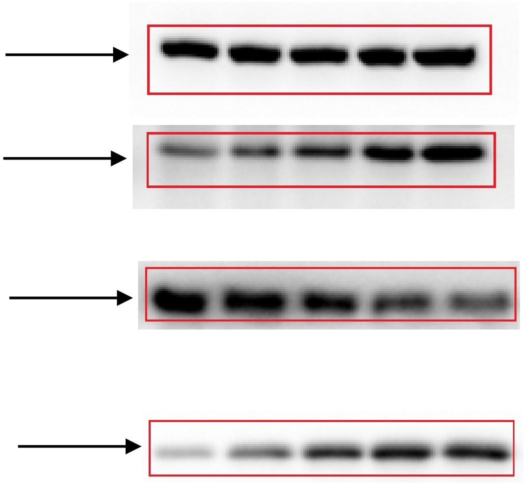

Full unedited blot for Figure 7C

The Full blot

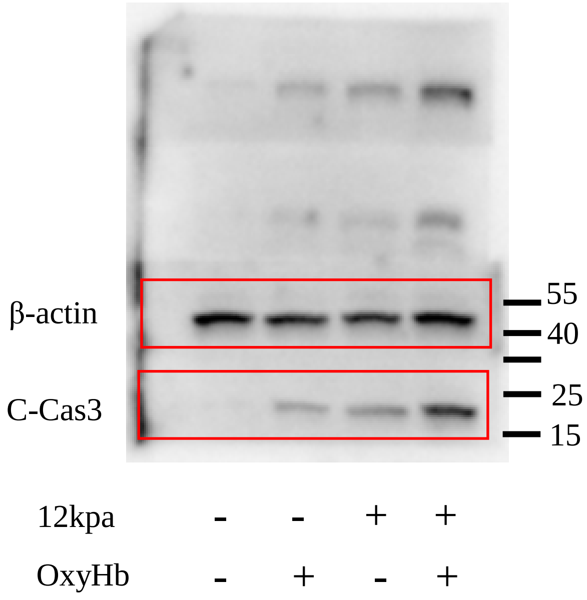

The cropped blot

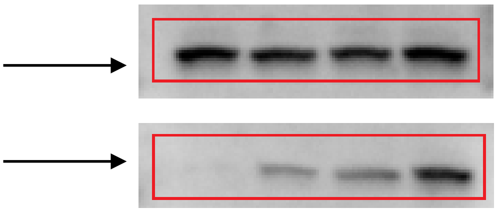

Full unedited blot for Figure 7C

The Full blot

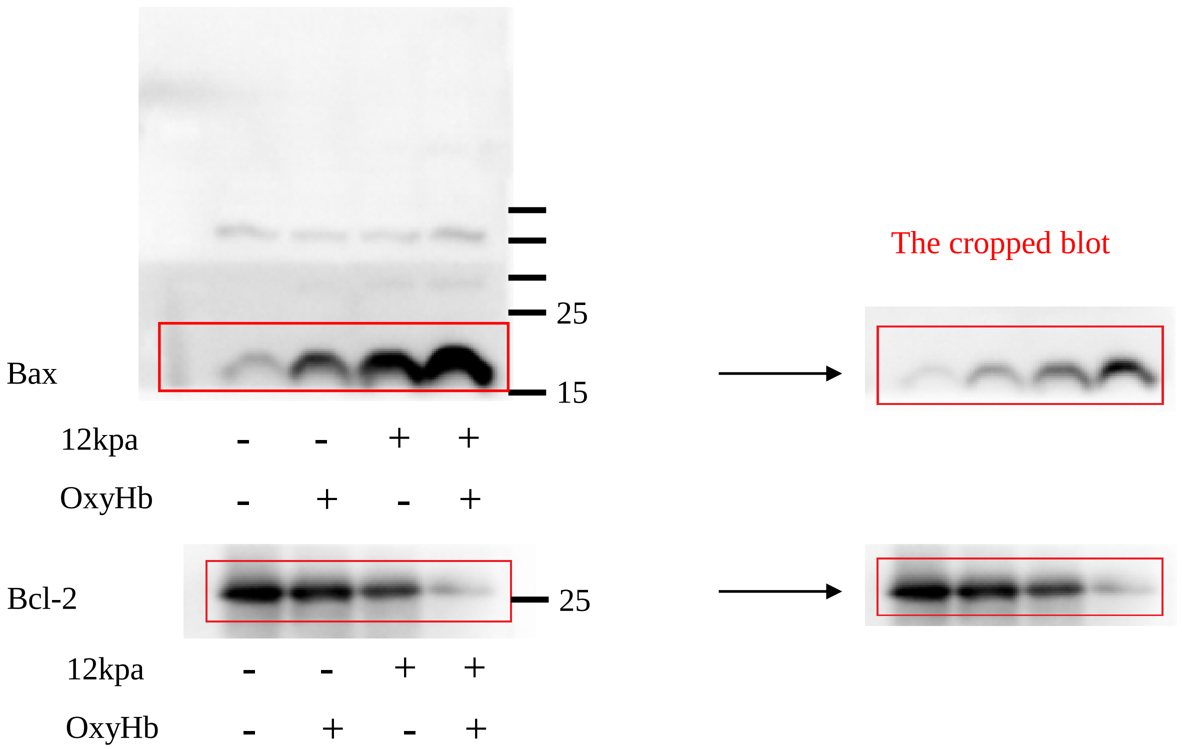

Full unedited blot for Figure 7F

The Full blot

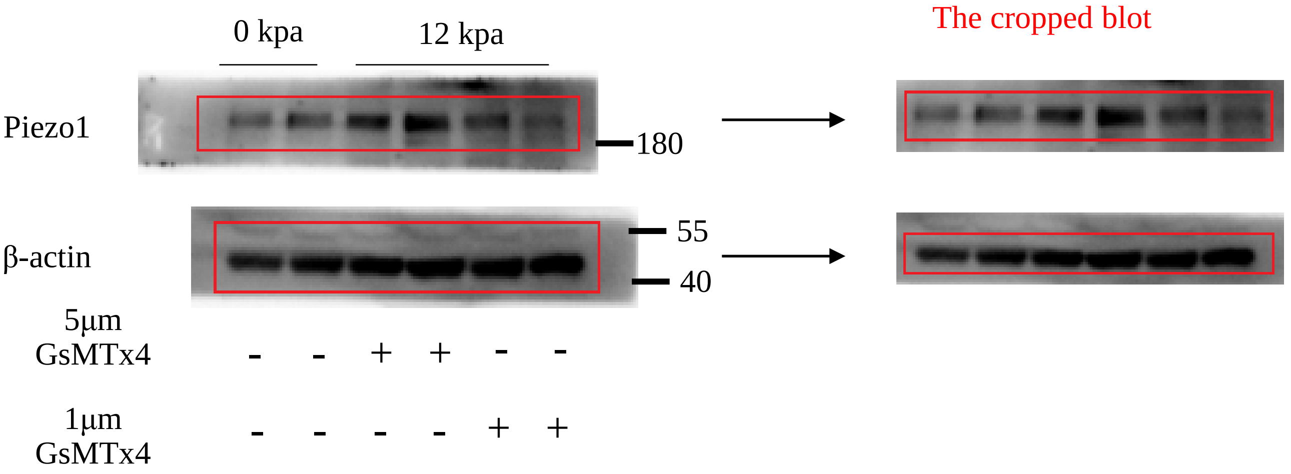

Full unedited blot for Figure 7H

The Full blot | The cropped blot

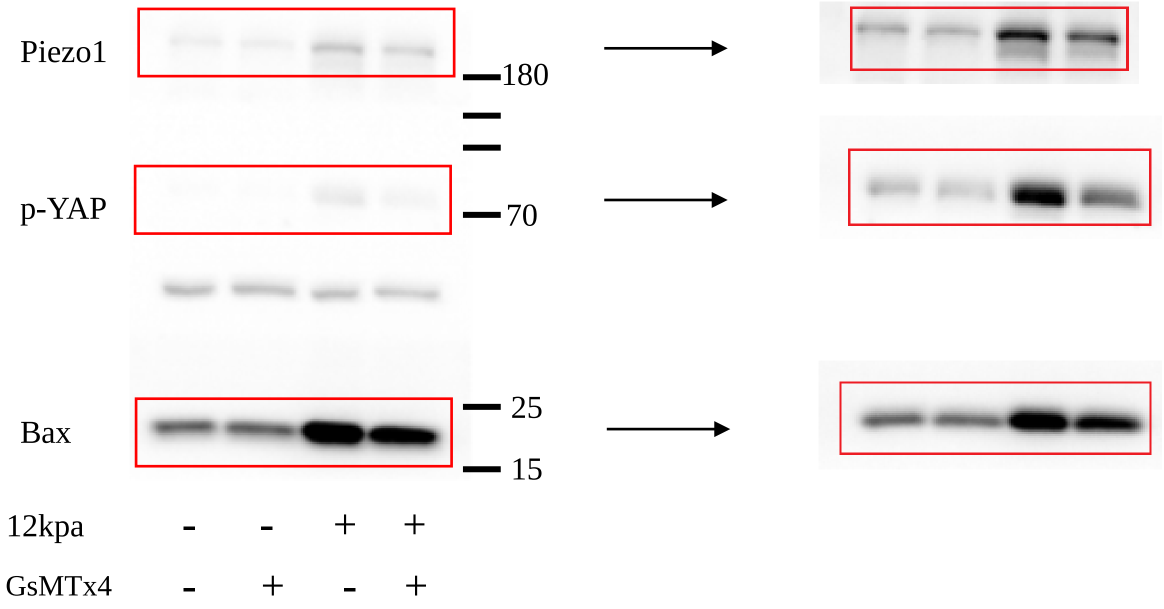

Full unedited blot for Figure 7H

The Full blot | The cropped blot

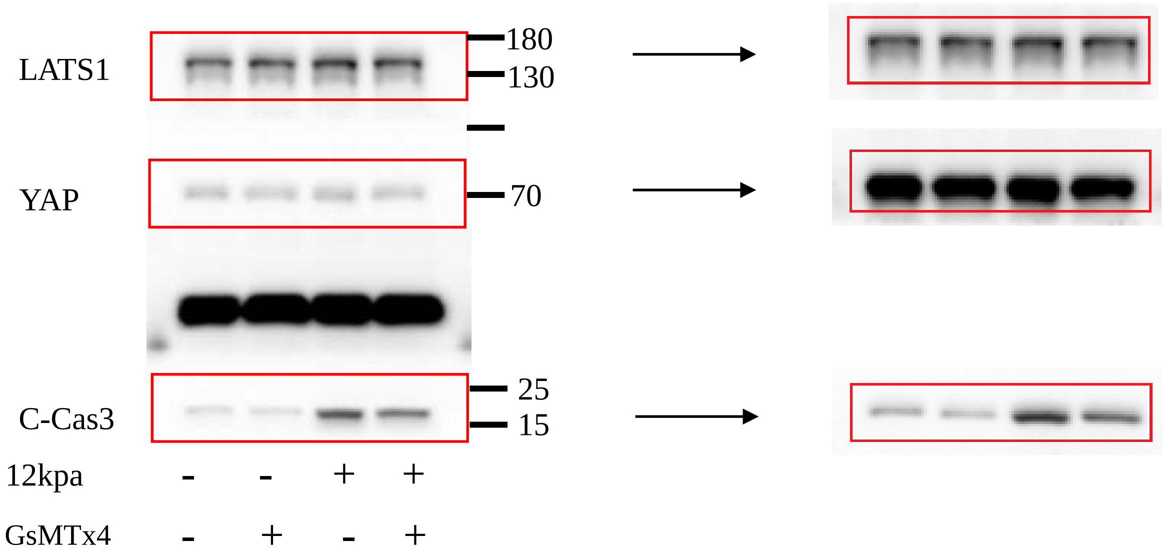

Full unedited blot for Figure 7H

The Full blot

The cropped blot

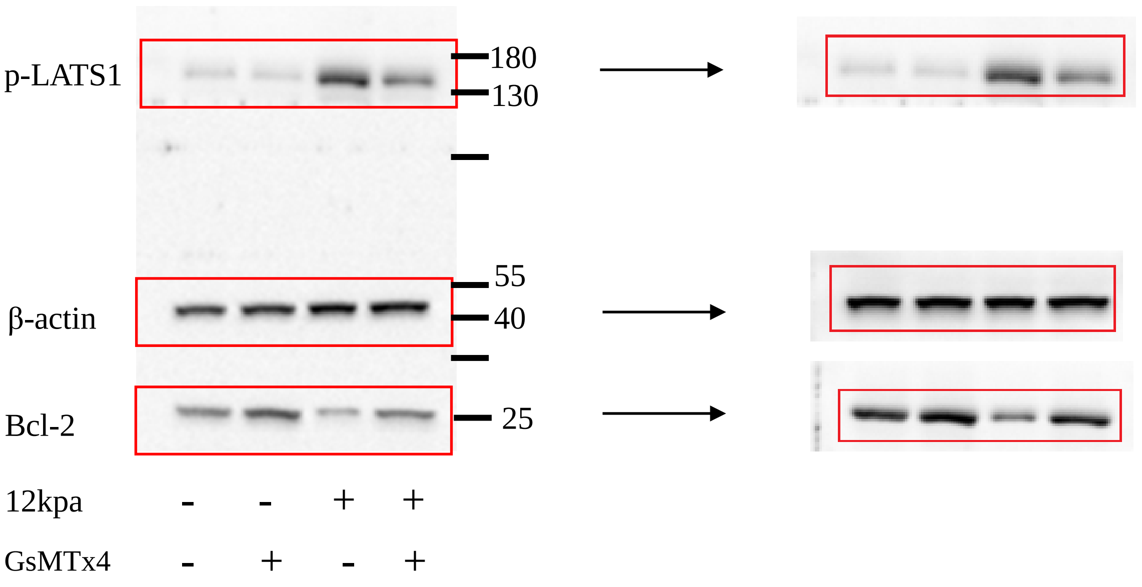

Full unedited blot for Figure 8A

The Full blot

The cropped blot

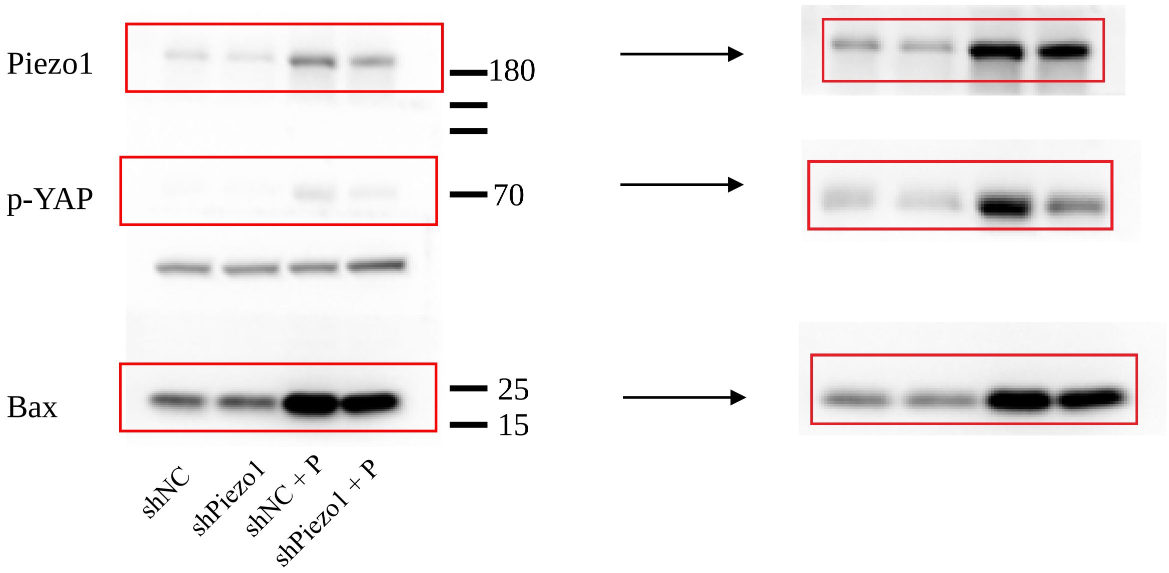

Full unedited blot for Figure 8A

The Full blot

The cropped blot

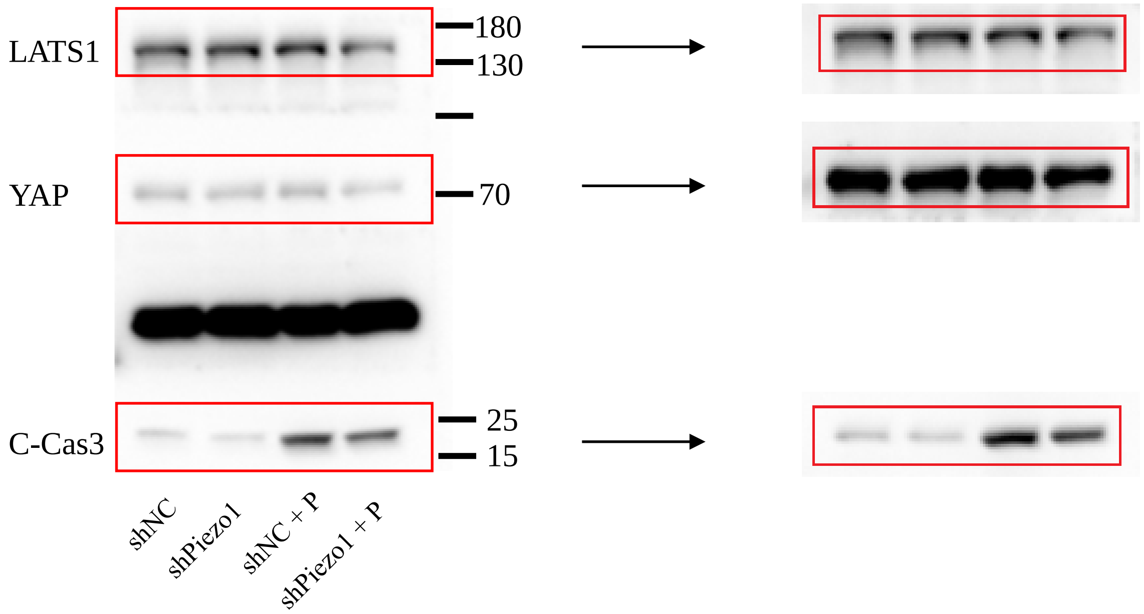

Full unedited blot for Figure 8A

The Full blot

The cropped blot

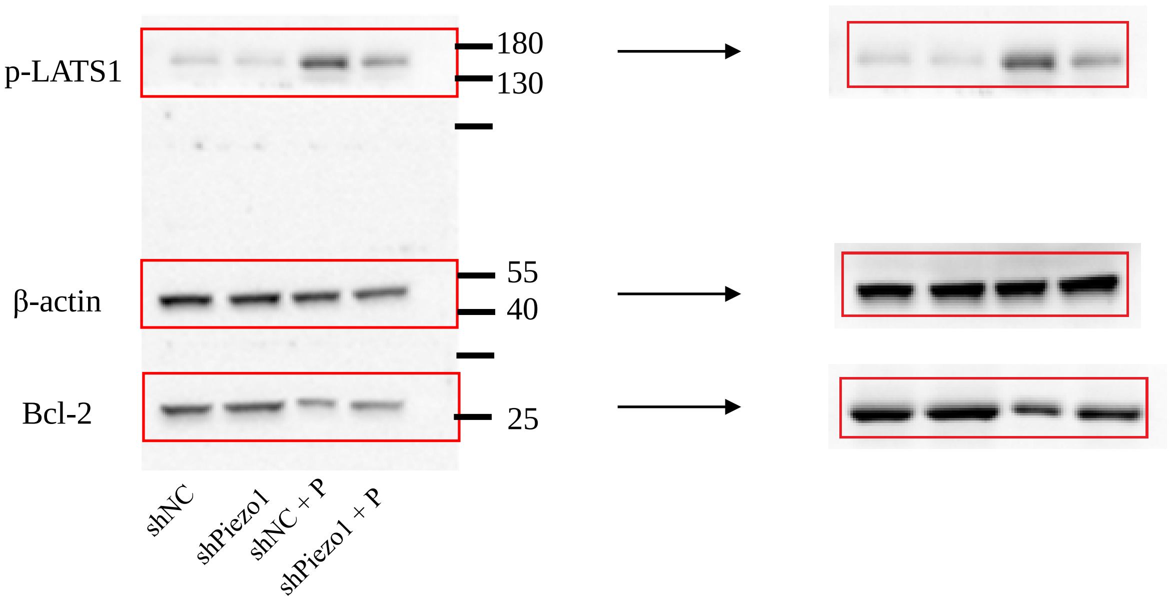

Full unedited blot for Figure 8C

The Full blot

The cropped blot

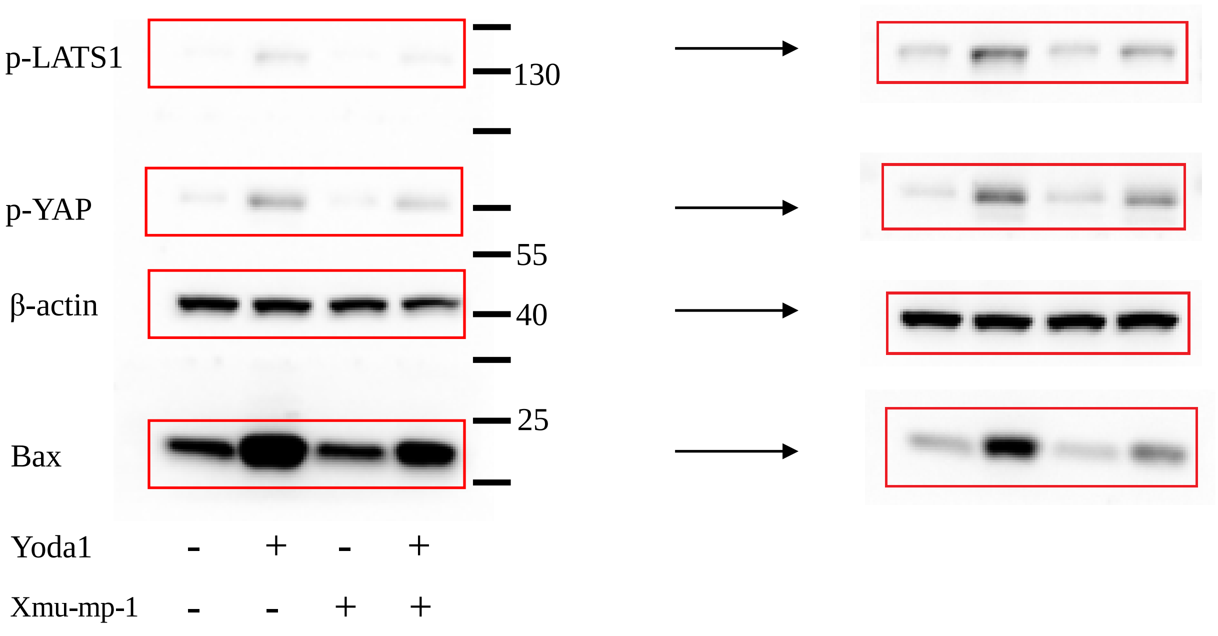

Full unedited blot for Figure 8C

The Full blot

The cropped blot

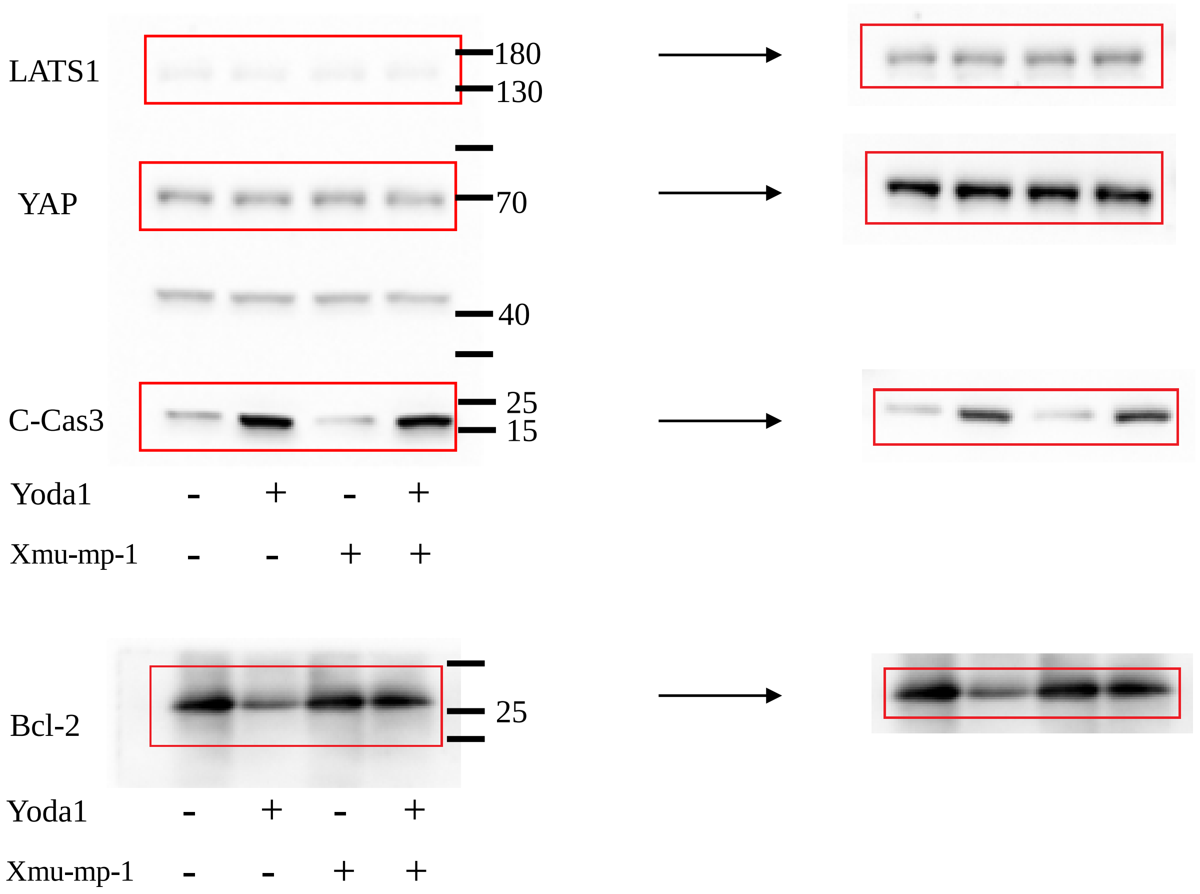

Full unedited blot for Figure S4B

The Full blot

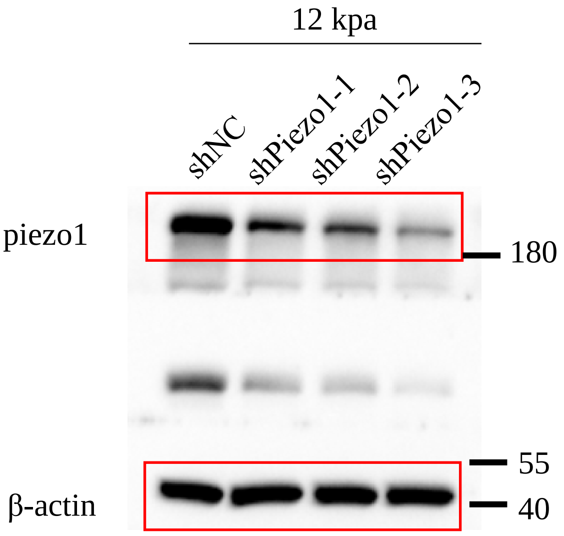

The cropped blot

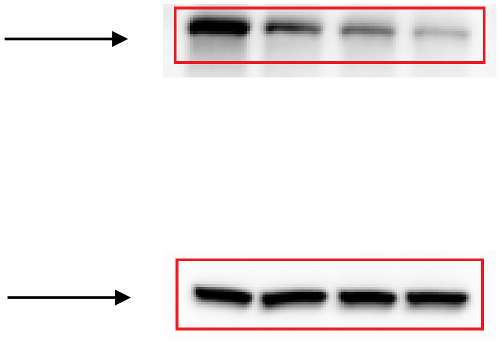

Supplement: Supplementary file 1 — Data S1. [file CNS-30-e14872-s002.pdf]
