# Supplementary figures and images for: Activation of Piezo1 by intracranial hypertension induced neuronal apoptosis via activating hippo pathway
Source: CNS Neurosci Ther. 2024 Sep 27;30(9):e14872. doi: 10.1111/cns.14872 (PMC11427798; doi:10.1111/cns.14872)

Figure S1


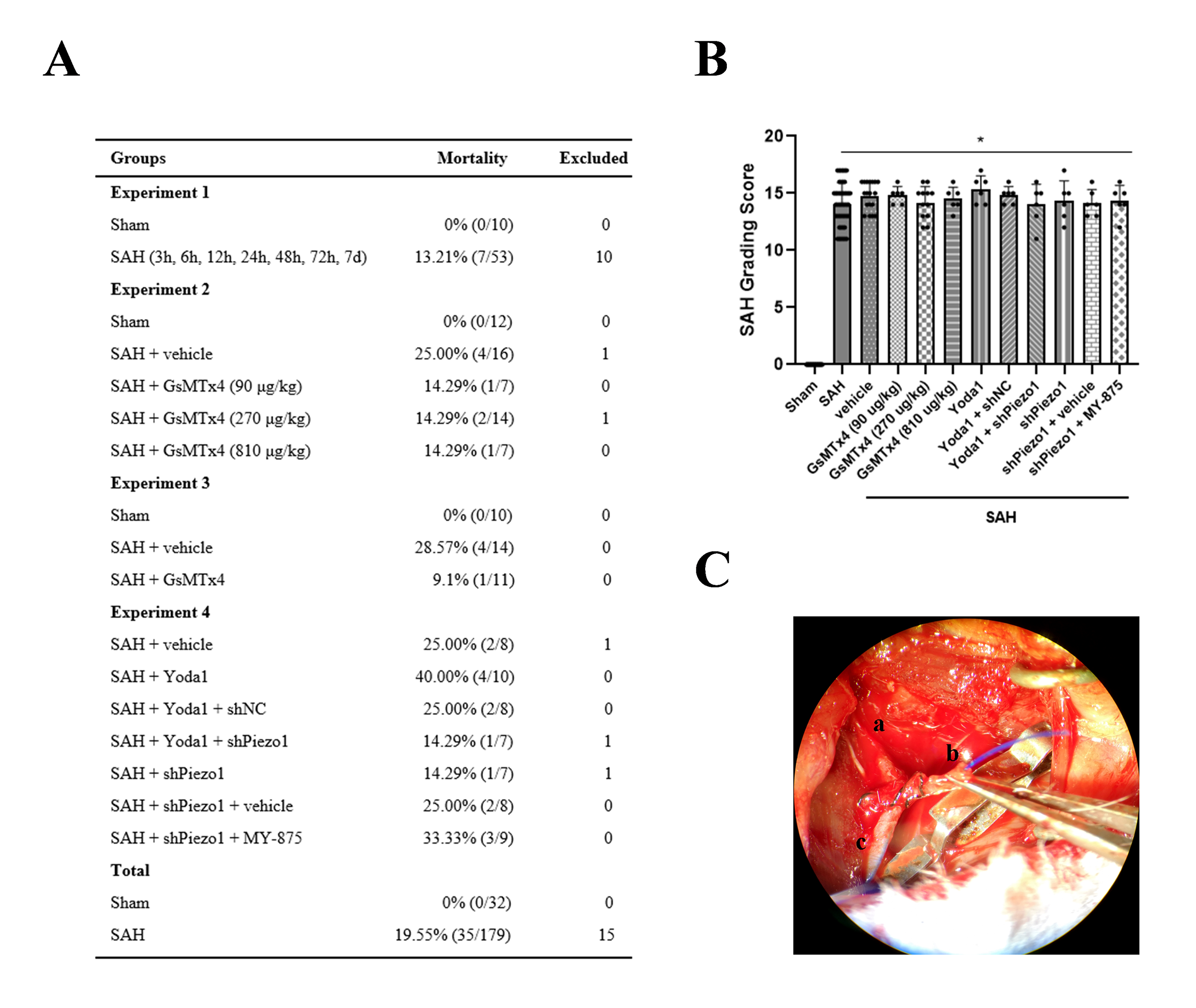


Figure S2


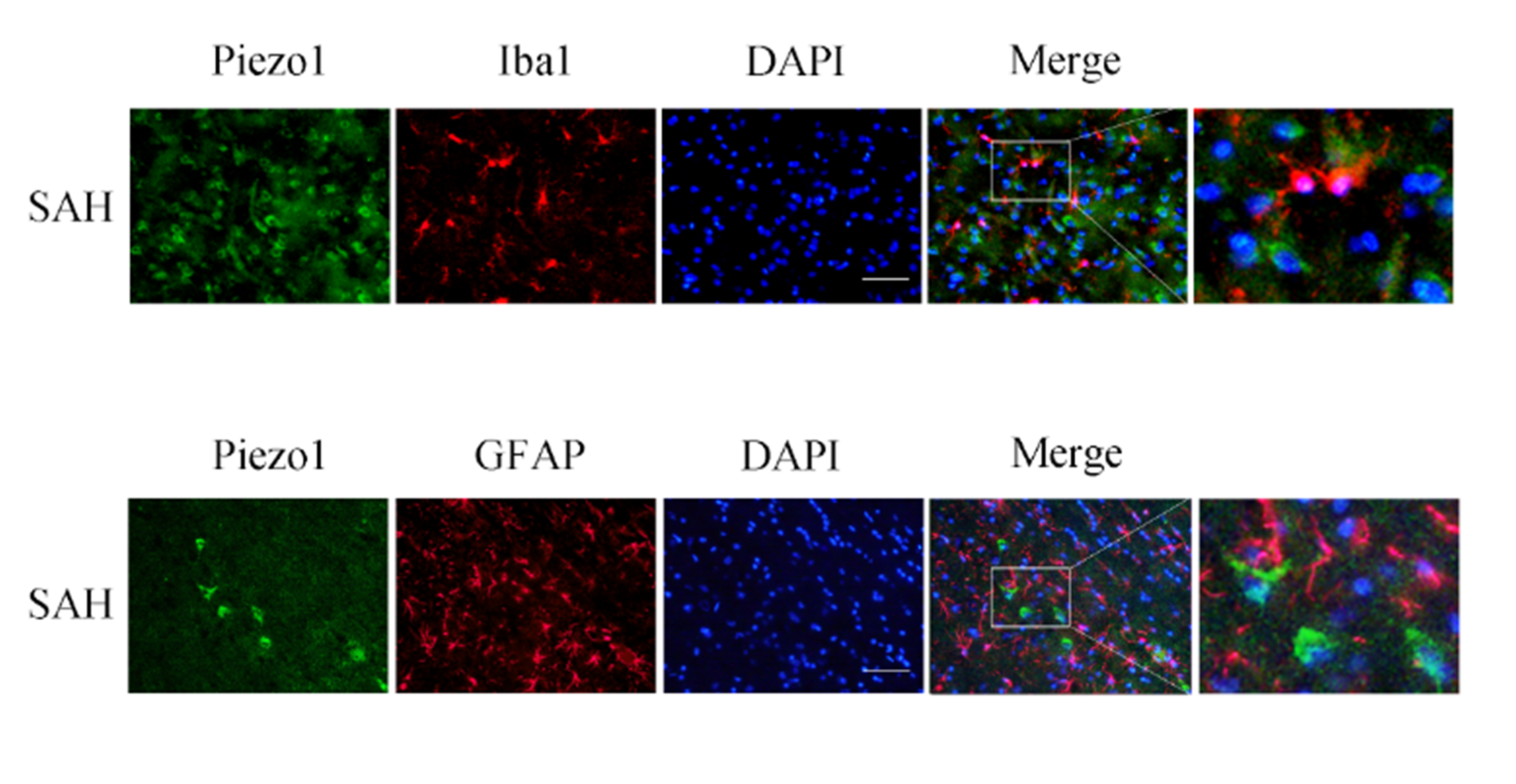


Figure S3


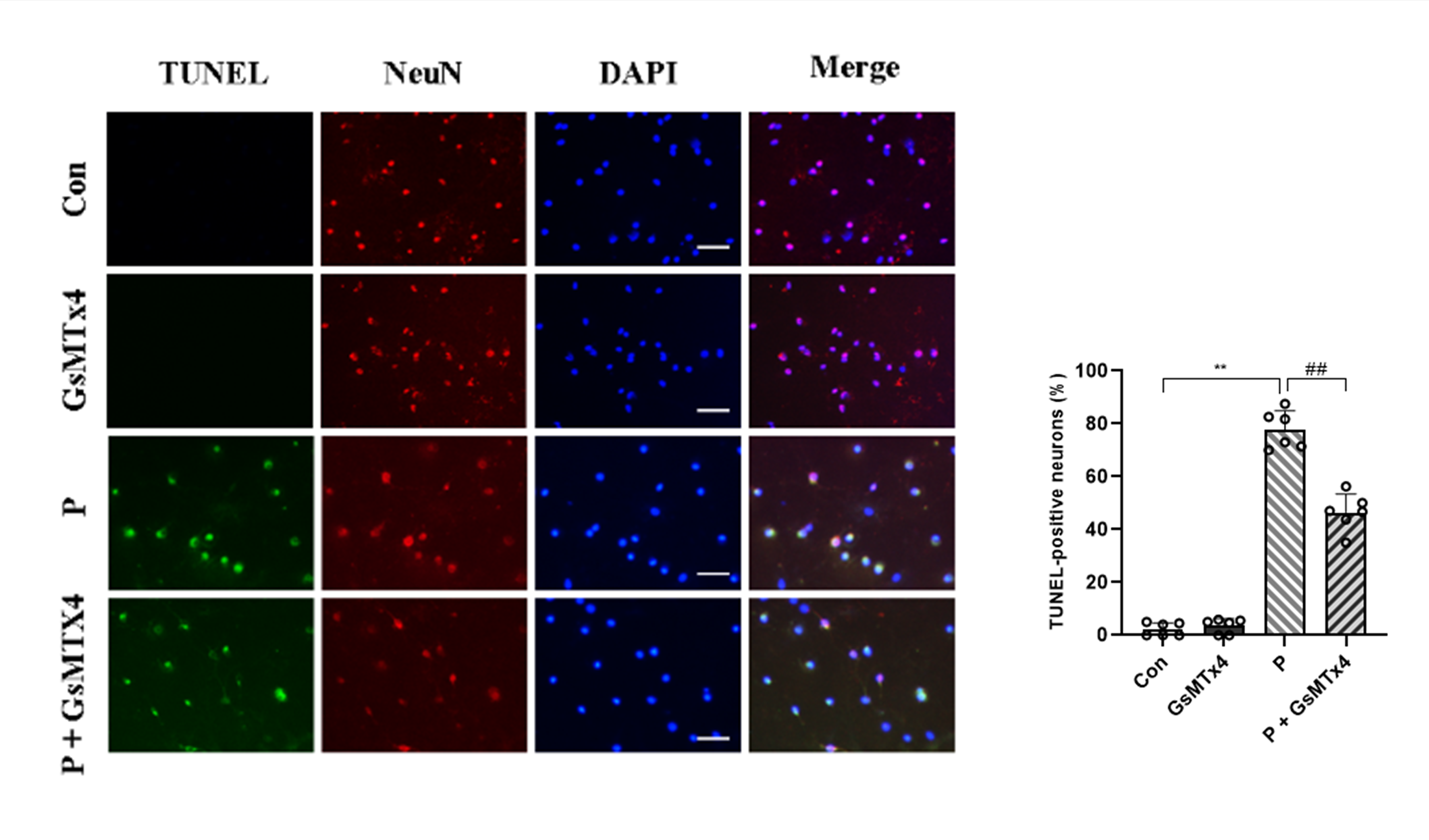


Figure S4


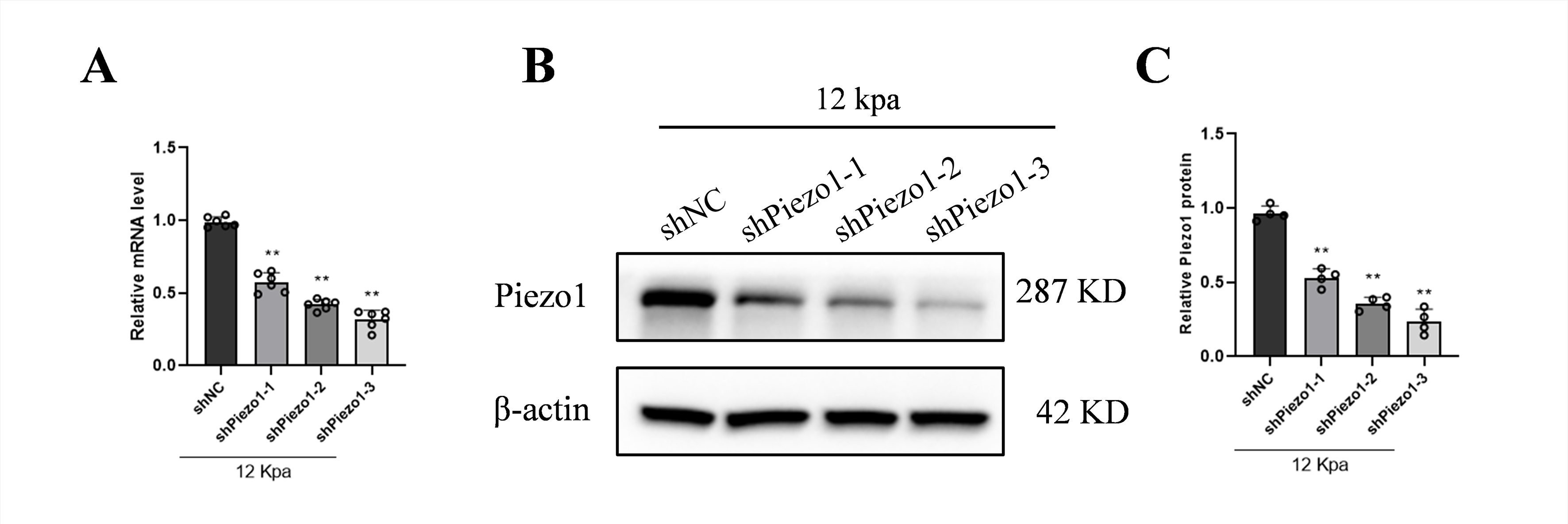

Supplement: Supplementary file 2 — Figures S1‐S4. [file CNS-30-e14872-s001.docx]
